# Combining modularity, conservation, and interactions of proteins significantly increases precision and coverage of protein function prediction

**DOI:** 10.1186/1471-2164-11-717

**Published:** 2010-12-20

**Authors:** Samira Jaeger, Christine T Sers, Ulf Leser

**Affiliations:** 1 Knowledge Management in Bioinformatics, Humboldt-Universitat zu Berlin Unter den Linden 6, 10099 Berlin, Germany; 2 Institute of Pathology, Molecular Tumorpathology, University Medicine Charite, Chariteplatz 1, 10117 Berlin, Germany

## Abstract

**Background:**

While the number of newly sequenced genomes and genes is constantly increasing, elucidation of their function still is a laborious and time-consuming task. This has led to the development of a wide range of methods for predicting protein functions in silico. We report on a new method that predicts function based on a combination of information about protein interactions, orthology, and the conservation of protein networks in different species.

**Results:**

We show that aggregation of these independent sources of evidence leads to a drastic increase in number and quality of predictions when compared to baselines and other methods reported in the literature. For instance, our method generates more than 12,000 novel protein functions for human with an estimated precision of ~76%, among which are 7,500 new functional annotations for 1,973 human proteins that previously had zero or only one function annotated. We also verified our predictions on a set of genes that play an important role in colorectal cancer (*MLH1*, *PMS2*, *EPHB4 *) and could confirm more than 73% of them based on evidence in the literature.

**Conclusions:**

The combination of different methods into a single, comprehensive prediction method infers thousands of protein functions for every species included in the analysis at varying, yet always high levels of precision and very good coverage.

## Background

Elucidating protein function is still one of the major challenges in the post-genomic era [1,2]. Even for the best-studied model organisms, such as yeast and fly, a substantial fraction of proteins is still uncharacterized [3]. As high-throughput techniques increase the availability of completely sequenced organisms, annotation of protein function becomes more and more a bottleneck in the progress of biomolecular sciences and the gap between available sequence data and functionally characterized proteins is still widening [2]. Manual annotation, using, for instance, the scientific literature, and experimental identification of protein function remains a difficult, time- and cost-intensive task [4]. Reliable methods for assigning functions to uncharacterized proteins are required to support and supplement these methods. There are various automatic approaches for the prediction of protein function. These use, for instance, protein sequences and 3D-structures [5-9], evolutionary relationships [10,11], phylogenetic profiles [12,13], domain structures [14], or functional linkages [15]. Another important class of information for function prediction are protein-protein interactions (PPIs). PPIs are a type of data that is close to the biological role of a protein within cells and therefore ideally suited to form the basis for function prediction methods [16,17]. Furthermore, more and more such data sets are becoming available (e.g. [18,19]). These data sets may be used to identify functional modules within protein networks [20], to find protein complexes [21], or to determine evolutionary conserved processes [22-25], all of which provide valuable clues to the function of a protein [3].

The approaches that use PPI for function prediction can be classified into two main classes:

1. Link-based methods predict novel functions for a protein by transferring known functions from directly or indirectly interacting proteins. This may be achieved by studying the set of neighbors [16,19,26,27], by considering the position of the protein within its neighborhood [28], or by looking at the position of the protein in the entire interaction network [29,30].

2. Module-based methods assign functions to proteins by first computing clusters (or modules) within the protein network [31]. Based on the hypothesis that cellular functions are organized in a highly modular manner [32,33], all members of a cluster are assigned annotations that are enriched within the module [23].

Both approaches have their benefits and their drawbacks. PPI-based prediction methods provide a better coverage but are sensitive to the high level of false-positives [34,35] and false negatives [36] in current PPI data sets. Module-based methods are more robust to missing or wrong interactions, but are able to predict function only within dense regions of a species network disregarding, for instance, chain-like pathways.  This largely reduces their coverage [21,31]. Module-based methods have been shown to be less accurate than for example simple guilt-by-association approaches but their performance improves in networks with less functional coverage [37]. Furthermore, both methods in first place only work within a species, which disregards the wealth of information that might be available in evolutionary related other species (this is particularly true for humans). This limitation can be removed by using annotations of homologous proteins. However, purely homology-driven prediction strategies are rather imprecise [38]. Although prediction precision may be improved by using only orthology, the overall precision remains below that of most PPI-based methods [7].

In this paper, we describe a novel algorithm for protein function prediction that combines link-based and module-based prediction with orthology, thus overcoming the respective limitations of each individual approach. The key to our method is to analyze proteins within modules that are defined by evolutionary conserved processes. To this end, we first compute PPIs that are highly conserved within a given set of species. These so-called interologs [39] are assembled to highly conserved protein sub-networks. For a given protein, we then predict functions of other proteins in the same CCS using both directly interacting proteins as well as orthology relationships.

We apply our function prediction strategy to different sets of species, ranging from species pairs to groups of up to four species. We show that our approach reaches very high prediction precision, especially for three and four species. Especially due to the combination of different sources of evidence for functional similarity between proteins, our method is able to predict many functions even for uncharacterized or only weakly characterized proteins. These functions are not reflected in the recall since these functions are novel, i.e., counted as FP in the comparison against a gold standard. For instance, when combining the novel predictions from different species combinations, we suggest 7,500 new functional annotations for 1,973 human proteins that previously had only zero or one function annotated. Overall, our method produces 12,300 novel annotations for human with an estimated precision of ~76% and 5,246 for mouse with ~81% precision. These numbers by far outreach that of comparable methods. It is also remarkable that our predictions are rather specific, which is reflected in a mean GO-depth of 8 for humans and 7 for mice. To confirm our estimated precision values, we manually verified a number of predictions in the context of colon cancer. Specifically, we studied the gene products *MLH1*, *PMS2 *and *EPHB4*, which received 14, 16, and 15 novel annotations through our method. Detailed literature analysis indicates that at least 73% of the novel functions actually are true predictions.

Finally, we compare our approach against three other approaches, *Neighbor Counting *[19], χ^2 ^[16], and *FS-Weighted Averaging *[27]. We show that our CCS-based method performs significantly better than those methods in almost all settings we studied, especially in terms of precision.

## Methods

**Figure 1:**
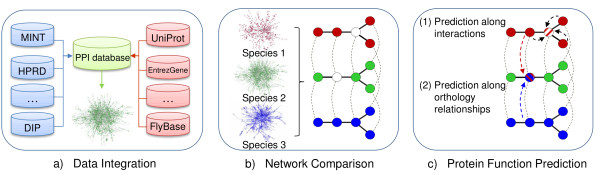
**Flowchart summarizing the main steps of our function method**. (a) We collect PPI data from several sources and integrate them with additional protein data to generate species-specific PPI networks. (b) PPI network comparisons are performed to identify CCS which (c) are analyzed afterwards for function prediction by exploiting orthology relationships and interacting neighbors.

We devise an algorithm for predicting functional annotations of proteins using Gene Ontology (GO) [40] terms. Our approach is based on comparison of interaction networks from various species and utilizes orthology relationships, conserved modules and local PPI neighborhoods. It is divided into the (a) integration of PPI data from various databases, (b) detection of maximal conserved and connected subgraphs (CCS) using approximate cross-species network comparisons and (c) prediction of new annotations for proteins within functionally coherent CCS (see Figure 1).

### Data

We use interaction data of the model organisms *S. cerevisiae*, *D. melanogaster *and *C. elegans*, and the mammals *R. norvegicus*, *M. musculus *and *H. sapiens*. Corresponding PPI data were obtained from the major public PPI databases DIP [41], IntAct [42], BIND [43], MIPS-MPPI [44], HPRD [45], MINT [46] and BioGRID [47]. Since the individual coverage and overlap between the data of these resources is comparably low [34,48], we integrate PPI data from the different sources to generate comprehensive data sets for our study. For data integration we map the interacting proteins from external or database specific identifiers to unique protein identifiers from UniProt and EntrezGene [49] to enable the combination of the different data sets to one comprehensive set of interaction data for each species. From the combined data sets we generated comprehensive species-specific protein interaction networks.

Besides the interaction data we utilize protein sequences and protein domain information [50] from UniProtKb/Swiss-Prot [51]. All proteins in the protein interaction network are associated with the respective information. Additionally, proteins are annotated with GO annotations retrieved from UniProtKb/Swiss-Prot, EntrezGene and species-specific databases, such as FlyBase [52], MGD [53], RGD [54], SGD [55] and WormBase [56] (see Additional File 1, Table S1 for a detailed resource listing). Note, when annotating proteins we consider all available GO annotations except for annotations that are assigned without curatorial judgment (GO evidence code: IEA - Inferred from Electronic Annotation). Moreover, we filter for GO subontology root terms to exclude molecular function, biological process and cellular component. The annotated species-specific protein interaction networks (see Table 1) provide the basis of our protein function prediction method.

**Table 1:** Characteristics of the generated species-specific PPI networks.

species	#proteins	#PPIs	GO terms/protein	median PPI/protein
*R. norvegicus *(*rno*)	973	1221	8	1
*M. musculus *(*mmu*)	3892	4670	4	1
*H. sapiens *(*hsa*)	13494	43637	2	2
*D. melanogaster *(*dme*)	10646	38723	3	3
*C. elegans *(*cel*)	3499	5858	1	1
*S. cerevisiae *(*sce*)	6578	67059	4	7

PPI networks for each species are created by integrating PPI data from DIP, BIND, IntAct, BioGrid, MIPS-MPPI, MINT and HPRD. Proteins within the networks are additionally associated with sequences, protein domains and GO annotations. For each species the number of proteins and protein interactions as well as the median number of GO terms per protein is specified.

### Network Comparison

**Figure 2:**
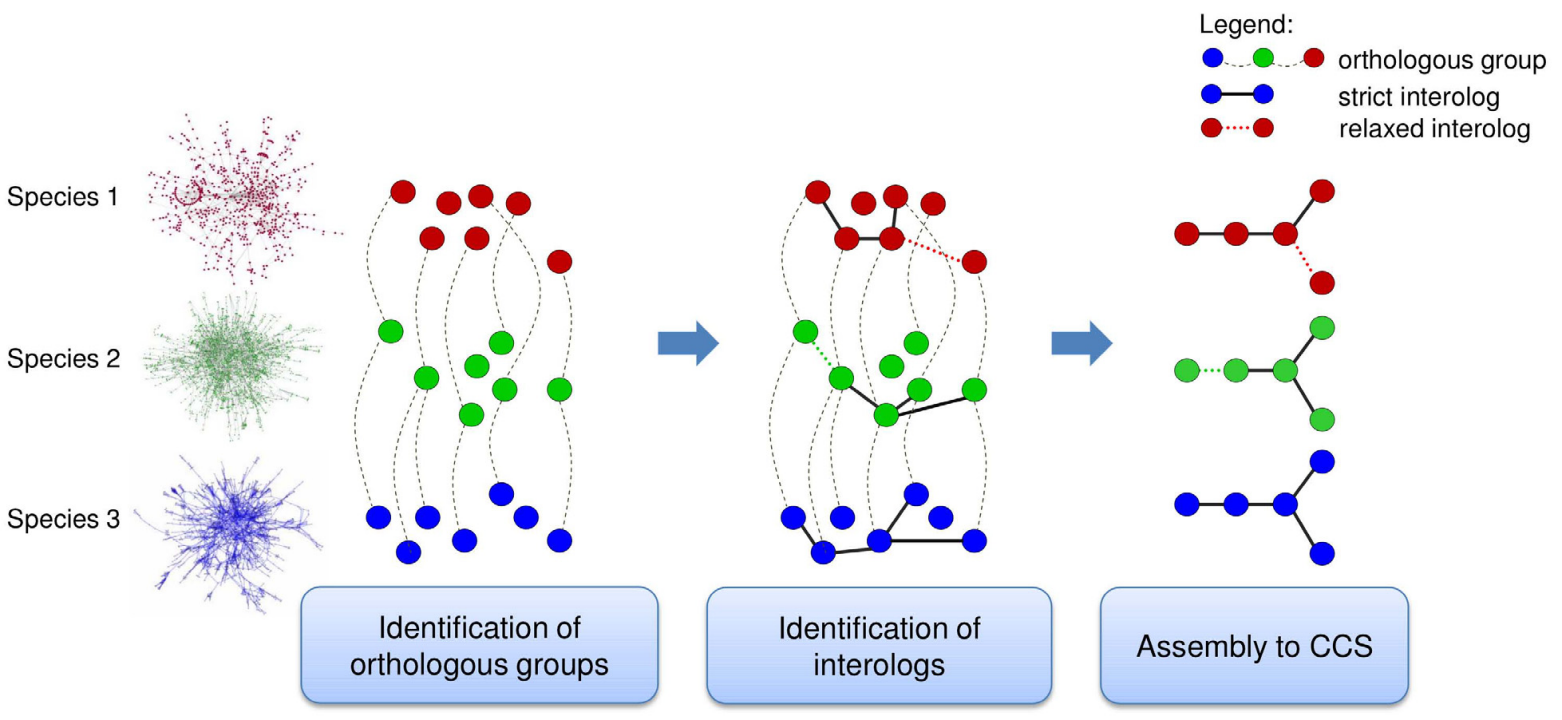
**Illustration of the detection of CCS**. Protein interaction networks are compared across different species to identify evolutionary and conserved subgraphs. First, orthology relationships across multiple species are determined by using OrthoMCL. Second, all pairs of conserved interactions (interologs) are identified between the orthologs within the species. Adjacent interologs are then assembled to CCS.

We compare protein interaction networks across different species to detect subgraphs that are evolutionary conserved and likely represent functional modules. Figure 2 depicts the strategy of our network comparison approach which involves (1) the identification of orthologous proteins and (2) the detection and assembly of interologs into CCS.

(1) Orthology is a strong indicator for functional conservation. However, the presence of large protein families, typical for mammals and higher eukaryotes in general, makes it hard to distinguish between true orthologs, in-paralogs and paralogs [57]. We determine orthology relationships among multiple species by applying OrthoMCL [58] using default parameters. Previous work showed that OrthoMCL is able to discriminate between orthologs, in-paralogs and functionally unrelated (out-)paralogs at a balanced trade-off between specificity and sensitivity [59].

(2) For comparing protein networks across species, we consider all ortholog groups that comprise at least one protein of each species under consideration. We then use an adaption of an algorithm for frequent subgraph discovery [60] to assemble interologs into CCS. Our approach first identifies all interactions (interologs) that are conserved across the different species. For identifying interologs we use two different definitions for interologs depending on the number of species that are involved. When comparing only two species, we use the classical, strict definition considering each interaction as interolog that is present in both species. When comparing more than two species, we consider each interaction as interolog that is present in more than 50% of the species networks (see Discussion). Out of the set of interologs, one interolog is chosen as subgraph seed and all interologs adjacent to this subgraph are added recursively. If a subgraph can not be further extended we store this maximal and connected subgraph as CCS (see Figure 2).

### Prediction of Functional Annotation

CCS are conserved subgraphs of interacting proteins and therefore a strong indicator for functional similarity of proteins within a CCS even across species. However, not all detected CCS are good candidates for function prediction due to the noise and incompleteness within the existing PPI and annotation data sets. Therefore, we first filter for CCS that are too heterogeneous or simply too small to be used for function prediction. We then use different methods for predicting functional annotations for all proteins in a CCS, namely transfer of annotations from other species along orthology relationships and transfer within species from all PPI neighbors. In both cases, only proteins within the same CCS are considered. Finally, special care has to be taken for the processing of large CCS which, due to their sheer size, usually are functionally heterogeneous. In the following, we give details for each of these steps.

#### Filtering coherent CCS

We first test all detected CCS for functional coherence using a functional similarity measure proposed by Couto *et al*. [61] that is based on semantic similarity. We compute, for each CCS, its average functional similarity within a species (*Sim_neigh _*- similarity between neighbors) and across the species (*Sim_ortho _*- similarity between orthologs). The formal definitions of both similarity measures are provided in the Additional File 1 (see Eq. S7 and S8 in Section S1.1).

We further only consider CCS which have (a) more than two proteins and (b) whose similarity score, either *Sim_ortho _*or *Sim_neigh_*, exceeds a given threshold. We applied three different thresholds (low: 0.3, medium: 0.5, high: 0.7) to study the performance of our method for different levels of functional coherence. This scheme is applied separately for each subontology of GO (molecular function (MF), biological process (BP), cellular component (CC)).

#### Prediction using orthology relationships

For inferring protein function from orthology relationships within a CCS, we determine orthologous groups that differ significantly in their individual functional similarity from the similarity score of the CCS by computing the standardized z-score (see Eq. S9). In groups with significant differences (p-value *<*0.01) we transfer all known protein annotations to poorly annotated or uncharacterized orthologs. Note that an orthologous protein group might consist of more than one protein per species (orthologs and in-paralogs). Although all proteins within such a group in theory should be functionally highly similar, this is, probably due to missing or wrong annotations, not always reflected in the data (see Results). We define the consensus annotation of all proteins of one species in an orthologous group to be the set of all GO terms that are associated to more than half of the annotated proteins of that species in that group. When considering more than two species we combine the species-specific sets of consensus annotations and transfer them to the other proteins in the same group.

#### Prediction using neighboring proteins

Given a protein in a CCS, we decide for each GO term annotated to any of its direct neighbors whether it also should be annotated to the protein itself. Let *G *be the set of terms annotated to at least one neighbor of a protein *p*, and let *N_g _*be the set of neighbors of *p *annotated with a term *g *∈ *G*. We transfer *g *to *p *if there are more than *f *proteins in *N_g _*whose functional similarity to *p *is higher than a given threshold *t*. For functional similarity between proteins, we again use the method from Couto *et al*. [61] (see Additional File 1, Eq. S5 in Section S1.1.2).

Because this approach cannot predict functions for proteins without any annotation (their computed similarity to other proteins is always zero), we also consider the pairwise functional relation between interaction partners, assuming that a high functional similarity between indirectly linked partners should also hold for the protein itself. Again, if the pairwise similarity scores exceed the threshold *t *we predict common GO annotations to the candidate protein.

#### Combined prediction method

We combine the two different methods to predict protein functions within a CCS (see Figure 1c). Proteins that are only weakly and incompletely characterized or not annotated at all are candidates for our prediction approach. For each candidate protein we infer novel protein function (a) within functionally coherent CCS by exploiting its (b) orthology relationship across other species as well as (c) the information shared by its neighboring proteins.

#### Processing large CCS

**Figure 3:**
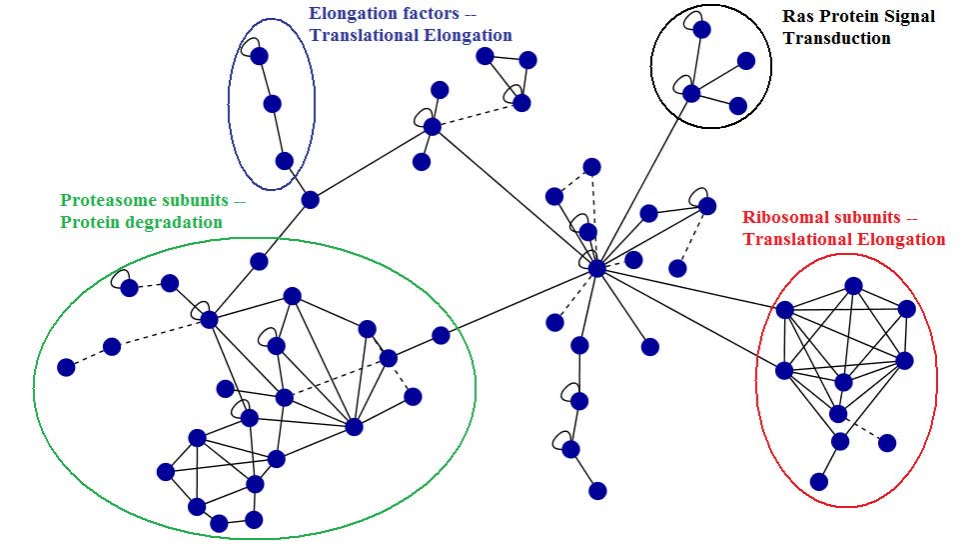
**Different biological subprocesses within the largest CCS from human, fly, worm and yeast**. This CCS consists of 61 proteins and 108 interologs and encompasses different biochemical activities, such as protein degradation, translational elongation and signal transduction.

Comparing evolutionary close species (such as human and mouse) often results in very large CCS with up to several hundreds of proteins. However, biological processes typically involve only between 5 and 25 proteins [21]. Consequently, large CCS often encompass various functions (see Figure 3) which is reflected in a minor functional homogeneity. Our results confirm this fact, as large CCS always get low coherence scores (see Results). To adequately treat such CCS, we split CCS with more than 25 proteins into smaller, overlapping sub-subgraphs. Sub-subgraphs are built by considering each protein of the CCS as seed of a new, smaller CCS. Subsequently, we add all direct neighbors of this seed to the new CCS (see Additional File 1, Figure S1 for an example). Subgraphs with less than three proteins are removed. We then consider each of these subgraphs as an independent CCS.

### Performance evaluation

We use a leave-one-out cross-validation to estimate the expected precision and recall of function prediction using (a) only orthology within CCS, (b) only neighbors within CCS, and (c) the combination of both methods. Precision *P *and recall *R *are defined as:

(1) $$P=\frac{\text{TP}}{\text{TP}+\text{FP}}$$

(2) $$R=\frac{\text{TP}}{\text{TP}+\text{FN}}$$

where TP and FP denote true and false positives, respectively, and FN denotes false negatives.

For cross-validation we 'hide' selected annotations before applying our algorithm. Predicted terms are then compared to the held out annotations. We count a GO term as correctly predicted if the proposed term was an ancestor of the original term on the path to the root or the term itself (see Additional File 1, Section S3.2 and Figure S2 for an evaluation of this criterion). For all methods involving CCS, we give recall values on the basis of all annotations of proteins within qualifying CCS. We call this measure per-protein recall. It must be distinguished from the traditional per-species recall (Eq. 2) which is also used frequently, but which punishes all methods that first filter proteins. When determining the per-protein recall (*R_pp_*) we consider only proteins *p *that are part of a CCS:

(3) $$R_{pp}=\frac{\sum_{p\;\in \;\text{C}\text{C}\text{S}}{\text{TP}}_{p}}{\sum_{p\;\in \;\text{C}\text{C}\text{S}}\left({\text{TP}}_{p}+{\text{FN}}_{p}\right)},$$

where TP*_p _*denotes the number of correctly predicted functions for a protein *p *in a CCS and (TP*_p _*+ FN*_p_*) corresponds to the number of annotations that are originally associated with the protein *p*. To also give an idea of the per-species performance, we always complement precision and recall values with the coverage measure, which simply counts the total number of predictions.

Keep in mind that, as always when comparing to an incomplete gold standard, cross-validation inherently considers any new annotations as false, although new annotations are the primary target of function prediction. Therefore, we also performed an extensive literature evaluation to judge the correctness of selected new annotations.

#### Comparison to other methods

We compare our approach against a number of different techniques.

First, we use two baseline methods: The orthology baseline purely considers orthology ignoring structural network conservation. We randomly select one third of the orthologous protein groups, remove annotations from one protein in the group and predict their functions using only its orthologs. The link-based baseline takes only direct interaction partners into account, again independently of conservation of interactions. For each species we randomly choose one third of the proteins from the corresponding interaction network and exploit their direct neighbors for deriving new functions. We repeat this procedure 100 times for each baseline and compute average and standard deviation across all runs.

We also compare our results with three popular PPI-based function prediction methods. The Neighbor Counting Approach from Schwikowski *et al*. is a local prediction approach that derives new annotations for a protein based on the frequency of annotations within its direct interaction partners [19]. The χ^2 ^algorithm from Hishigaki *et al*. extended this idea by also considering the background frequency of a functional term [16]. Finally, the Functional Similarity Weighted Averaging method from Chua *et al*., a weighted averaging method to predict the function of a protein based on its direct and indirect interaction partner [27,62]. Chua *et al*. demonstrate in [27] that the FS-Weighted Averaging significantly outperforms local and global network approaches, e.g. methods that are based on markov random field or functional flow [26,29]. For comparisons, we adapted a script provided by Chua *et al*. that implements these three methods (see Additional File 1, Section S1.4 for details). To enable a valid direct comparison, we evaluate the three related predictions methods only on proteins that are involved in CCS. The individual performance of each method on the entire data set is shown for completeness in the Additional File 1.

## Results

We integrated PPI data for rat (*rno*), mouse (*mmu*), human (*hsa*), fly (*dme*), worm (*cel*) and yeast (*sce*) from several public databases to generate species-specific PPI networks (see Table 1). We computed CCS for 15 combinations of two species, 20 comparison with three, and 11 with four species, and subjected them to our function prediction method. The number of detected CCS for combinations of five and six species is too low for a systematic and detailed analysis (see Additional File 1, Table S2).

In the following, we focus on four selected species combinations that cover different interactome sizes and evolutionary distances to discuss properties and results of our function prediction strategy. Complete results are given as Additional File 2, Table S2 and Additional File 3, Table S3.

### Network Comparisons

We compared protein interaction networks across different species to identify evolutionary and functionally conserved subgraphs that are used as basis for function prediction. Conserved sub-networks are assembled by combining conserved interactions, called interologs, using different definitions of interologs depending on the number of species being compared. For species pairs, we use the classical, strict definition: An interolog is an interaction present in both species. We relax this demand when comparing more than two species to cater for evolutionary variation [63] and for the incompleteness [36] and noise within present PPI data sets [34]: An interolog then is defined as an interaction which is present in more than 50% of the species being compared.

We present a brief overview on the respective network comparison of *rno-dme*, *rno-hsa-sce*, *hsa-dme-sce*, *hsa-dme-cel-sce *and *mmu-hsa-dme-cel *(see Additional File 2, Table S2 for complete results). Table 2 summarizes the outcomes for the selected species combinations in terms of orthologous protein groups, identified interologs and assembled CCS. As expected, the number of orthologous protein groups, interologs and identified CCS differs depending on the number of compared species, their evolutionary distance as well as their current interactome coverage. Comparison of fly and yeast results in 17 CCS (out of 73) with at least three proteins. For more than two species we use the relaxed interolog definition which generally results in a considerable higher number of CCS. For instance, we identify 163 CCS for *hsa-dme-sce *of which 23 comprise more than two proteins. These CCS are shown in Additional File 1, Figure S3. Even combinations with four species result in a reasonable number of CCS, such as *mmu-hsa-dme-cel *producing 16 CCS with more than two proteins.

**Table 2:** Overview on the outcomes of the selected network comparisons.

	# OrthoMCL groups	# Interologs	# CCS (≥3)	largest CCS
*dme-sce*	1514	137	73(17)	17

*rno-hsa-sce*	151	88	31(9)	22

*hsa-dme-sce*	542	692	163 (23)	187

*hsa-dme-cel-sce*	519	300	94 (4)	61

*mmu-hsa-dme-sce*	325	238	73 (16)	20

For each species combination the number of orthologous groups, interologs, CCS are presented as well as the size of the largest CCS. Note, we use the strict interolog definition for two species and the relaxed criterion for multiple species (see Methods).

### Function Prediction

We use orthology relationships, functionally conserved modules, and direct and indirect protein interactions for predicting functional annotations for proteins in a CCS by transferring annotations from other species along orthology relationships and within species from interaction partners. We evaluated our approach in three ways. First, we compared our combined strategy to baseline methods which disregard conservation in networks. Second, we compared it to the results obtained from using orthology and PPI neighborhood within CCS in isolation. Third, we performed a comparison to three recent function prediction methods from the literature.

We first show the performance of our two baseline methods, orthology and link-based, for function prediction. Precision for predictions based solely on orthology relationships varies between 3% and 11% (see Additional File 1, Table S4). Recall is higher (3% to 40%), but decreases steeply with the number of species being compared. Precision of the link-based baseline ranges from 3% to 17%. Contrary to the orthology baseline, recall is rather high, varying between 51% and 75% (see Additional File 1, Table S5). Thus, the link-based baseline reaches a similar precision but higher recall than the orthology baseline. Both baselines yield very low precisions. The orthology baseline indicates the challenges transferring function from ortholog templates. Although function tends to be conserved in orthologs, orthology does not guarantee conservation of function [38]. When transferring function solely based on protein sequences, more sophisticated approaches, e.g. using advanced statistical frameworks [9], are needed to ensure high prediction quality. The precision of the link-based baseline is lower than expected most likely through the strong impact of the quality of the interaction data. However, precision and recall are similar to the results of the two local prediction approaches of Schwikowski *et al*. and Hishigaki *et al*. that are applied to our data (see Discussion).

#### Across Orthology Relationships within CCS

We use orthology relationships underpinned by interologs to infer novel functions from multiple species. Considering only orthology relationships for transferring functions to proteins within CCS results in predictions with medium to high precision. Additional File 1, Table S6 shows precision and recall estimated using cross-validation for the selected examples. Precision reaches 88% to 97% for yeast proteins when comparing *hsa-dme-sce *and 67% to 85% for mouse proteins when comparing *mmu-hsa-dme-sce*. Precision values increase considerably with a higher coherence threshold for CCS, but this improvement comes at the cost of lower coverage. Particularly low numbers of predictions are obtained for comparisons involving species with low PPI coverage. This is especially prominent for *rno*, where comparison of *rno-hsa-sce *result in only 8 predictions - but with a precision of 100%.

Besides the coherence threshold, also the number of species being compared has a strong impact on performance. Higher average precisions are achieved when analyzing multiple species compared to species pairs. For instance, the average precision for *mmu-hsa-dme-sce *is 79% at 0.3 in comparison to *dme-sce *with 54% at 0.3 and 69.5% at 0.7. This shows that using more species implicitly selects functions that are conserved more strongly, which underlines the impact of evolutionary functional conservation for protein function prediction. This fact also shows up when comparing to the orthology baseline (see Additional File 1, Table S4): Precision and per-protein recall using orthology within CCS are much higher, but the overall coverage is much lower. This means that CCS strongly restrict the number of proteins for which predictions are made, but this restriction is done in a very sensible way removing mostly false positive predictions.

### Across Neighborhood within CCS

Additional File 1, Table S7 shows precision and recall for inferring functions only from interaction partners within CCS. Compared to predicting function based on orthology within CCS, precision is higher, while per-protein recall roughly stays the same. At the same time, neighbor-based prediction has a considerable better coverage. However, there are also species combinations in which this method performs worse. Precision again correlates with the functional coherence of CCS and with the number of compared species, but the impact is less pronounced. Especially the step from coherence threshold 0.3 to 0.5 mostly makes only a small difference. Compared to the link-based baseline (see Additional File 1, Table S5), precision is much higher and coverage and per-protein recall decreases.

#### Combining module, orthology and link-based PPI evidences

We hypothesized that the integration of orthology relationships, evolutionary conserved functional modules, and direct and indirect protein-protein interactions into a single prediction strategy will combine the strengths of the three individual methods. Selected results from this combined strategy are shown in Table 3 (see Additional File 3, Table S3 for complete results). As before, precision varies (from 46% to 91%) depending on the species combination and the threshold for functional coherence of CCS. Best results are obtained for *rno-hsa-sce *at a threshold of 0.7, with precision of 85%, 89% and 86%, respectively.

**Table 3:** Prediction results when combining module-based CCS, orthology relationships, and neighboring proteins.

		0.3			0.5			0.7	
	# terms	P	**R**_***pp***_	# terms	P	**R**_***pp***_	# terms	P	**R**_*pp*_
*dme*	6242	0.50	0.29	5072	0.52	0.25	1522	0.73	0.32
*sce*	3567	0.61	0.27	2581	0.71	0.28	1303	0.83	0.40

*rno*	1125	0.63	0.20	485	0.67	0.27	1185	0.85	0.30
*hsa*	1489	0.56	0.29	368	0.85	0.34	223	0.89	0.34
*sce*	1870	0.60	0.25	1206	0.61	0.17	229	0.86	0.24

*hsa*	13975	0.46	0.35	4418	0.57	0.36	723	0.73	0.33
*dme*	18638	0.62	0.41	16225	0.61	0.38	3462	0.71	0.48
*sce*	16544	0.72	0.44	15524	0.72	0.43	4135	0.84	0.55

*hsa*	3314	0.47	0.25	439	0.75	0.28	160	0.91	0.41
*dme*	5190	0.58	0.22	4586	0.59	0.23	866	0.81	0.29
*cel*	2464	0.47	0.27	1796	0.56	0.27	256	0.65	0.31
*sce*	5361	0.70	0.31	5126	0.71	0.32	1212	0.80	0.37

*mmu*	1212	0.66	0.17	459	0.81	0.32	53	0.81	0.34
*hsa*	3301	0.48	0.28	1658	0.57	0.33	436	0.65	0.81
*dme*	5561	0.56	0.29	4642	0.57	0.29	1400	0.59	0.55
*sce*	5159	0.63	0.31	4906	0.63	0.31	2140	0.73	0.72

average	5870	0.58	0.29	4343	0.65	0.30	1160	0.77	0.42

Precision (P) and per-protein recall (Rpp) are estimated for low (0.3), medium (0.5) and high (0.7) functional similarity/conservation thresholds.

As mentioned before, one of the major drawbacks of using only CCS orthology relationships is the low number of predictions due to the restriction to orthologous proteins with at least one known function (see Additional File 1, Table S6). In contrast to orthology-only, the combined approach creates many more predictions (2- to 50-times more). It generates hundreds or even thousands of predictions also for those cases where the orthology-only method could not predict any function.

Comparing the combined method and CCS link-based only (see Additional File 1, Table S7) shows an increase within the amount of predictions (e.g. about 2-times for *dme *from *dme-sce*), although it is less steep than observed for orthology-only. This increase has mostly only minor influence on precision and recall. Precision reaches similar levels and the recall increases slightly. Note, for few combinations the combined method yields the same results as link-based-only because no predictions could be inferred through orthology relationships.

Overall, the impact of our combined approach is dominant, especially in terms of the number of predictions. Precision drops for some combinations compared to the single methods. However, the decrease of precision does not indicate a lower prediction quality. It rather indicates that the combined method derives many more novel predictions that can not be validated during cross-validation rather than successfully reproducing known function for well-characterized proteins (see Discussion of predictions). Precision is affected the least for the highest similarity threshold (0.7) fostering the most reliable precisions.

#### Overlap between orthology- and link-based predictions within CCS

We combined orthology- and link-based function prediction within CCS to benefit from the strengths of both methods. To study whether the predictions of the individual methods result in the same or complementary sets of predictions we determined the overlap of GO terms predicted by either strategy. For *hsa-dme-sce*, the respective numbers are shown as Venn diagrams in Additional File 1, Figure S4. In general, the major fraction of unique predictions is derived from neighboring proteins. The overlap between predictions is comparably small and decreases when increasing the similarity threshold. This shows that both methods complement each other very well as they predict rather different sets of functions. For *hsa-dme-sce*, the respective numbers are shown as Venn diagrams in Additional File 1, Figure S4. In general, the major fraction of unique predictions is derived from neighboring proteins. The overlap between predictions is comparably small and decreases when increasing the similarity threshold. This shows that both methods complement each other very well as they predict rather different sets of functions.

This behavior is also observable when predictions are analyzed separately per species (see Additional File 1, Figure S5). However, contrary to fly and yeast proteins (see Additional File 1 Figure S5(b) and S5(c)), the amount of orthology and link-based predictions is quite similar for human proteins (see Additional File 1, Figure S5(a)), which can be explained by the much denser PPI data available for the two model organisms (see Table 1). This observation clarifies that different species pro t differently from our method. Especially less characterized species, such as human, benefit strongly from the functional knowledge of model organisms.

#### Overlap between predictions derived from different species combinations

Not only does the neighbor-based method complement the orthology-based method, but also predictions derived from different species combinations are rather complementary. Table 4 shows the overlap between predictions for human proteins inferred from different species pairs. The overlap is determined by dividing the number of overlapping predictions through the total number of predictions of a combination (expressed as percentage). The overlap mostly is far below 50% and strongly depends on evolutionary distance between the species. For example, the overlap between predictions derived from CCS with mouse and those derived from rat is much larger than that of the sets derived from mouse and, say, fly. The same holds for combinations of three and four species (data not shown). Moreover, the more species we combine the more we focus our prediction on evolutionary conserved functions, which becomes clear when studying predictions for highly conserved housekeeping functions (see Discussion).

**Table 4:** Fraction of overlapping function predictions (in %) for human proteins derived from different species pairs.

	*mmu-hsa*	*hsa-dme*	*hsa-cel*	*hsa-sce*
*rno-hsa*	44.6/48.7	40.4/14.1	22.0/28.0	33.3/19.4
*mmu-hsa*	-	47.7/16.1	21.5/94.4	52.4/25.1
*hsa-dme*	-	-	4.2/17.5	39.4/42.9
*hsa-cel*	-	-	-	89.0/74.2

The overlap is defined as the number of overlapping predictions divided by the total number of predictions (expressed as percentage). Each cell contains two different values - i/j - that specify the overlap based on the total number of predictions of the two combinations. i presents the overlap between the non-human species from row i and column j and value j presents the overlap between non-human species from column j and row.

#### Large CCS

Large CCS naturally encompass various biological functions. In consequence, their functional homogeneity is often too low which excludes the entire CCS from function prediction. However, large CCS actually are strong indicators for conserved functions. For instance, Figure 3 shows the largest CCS from *hsa-dme-cel-sce *consisting of 61 proteins and 108 interologs with its different biological subprocesses. It clearly contains several functionally highly conserved clusters, probably forming discrete protein complexes. Considering such a large CCS as a whole is insufficient. Therefore, we modify our approach for large CCS by breaking them up into sub-subgraphs (see Methods). The impact on precision and recall is shown in Additional File 1, Table S8 (large CCS are split), which should be compared with entries of Table 3 (large CCS are ignored). As can be seen, processing large CCS creates many more predictions with mostly better precision. For example, the number of predictions almost triples for *hsa-dme-sce *at a similar or even better precision. When comparing split and non-split results from *hsa-dme-cel-sce *the precision decreases for human along a five-fold increase of the number of predictions, but increases for all the other species (at 0.7).

### Comparing with other methods

**Figure 4:**
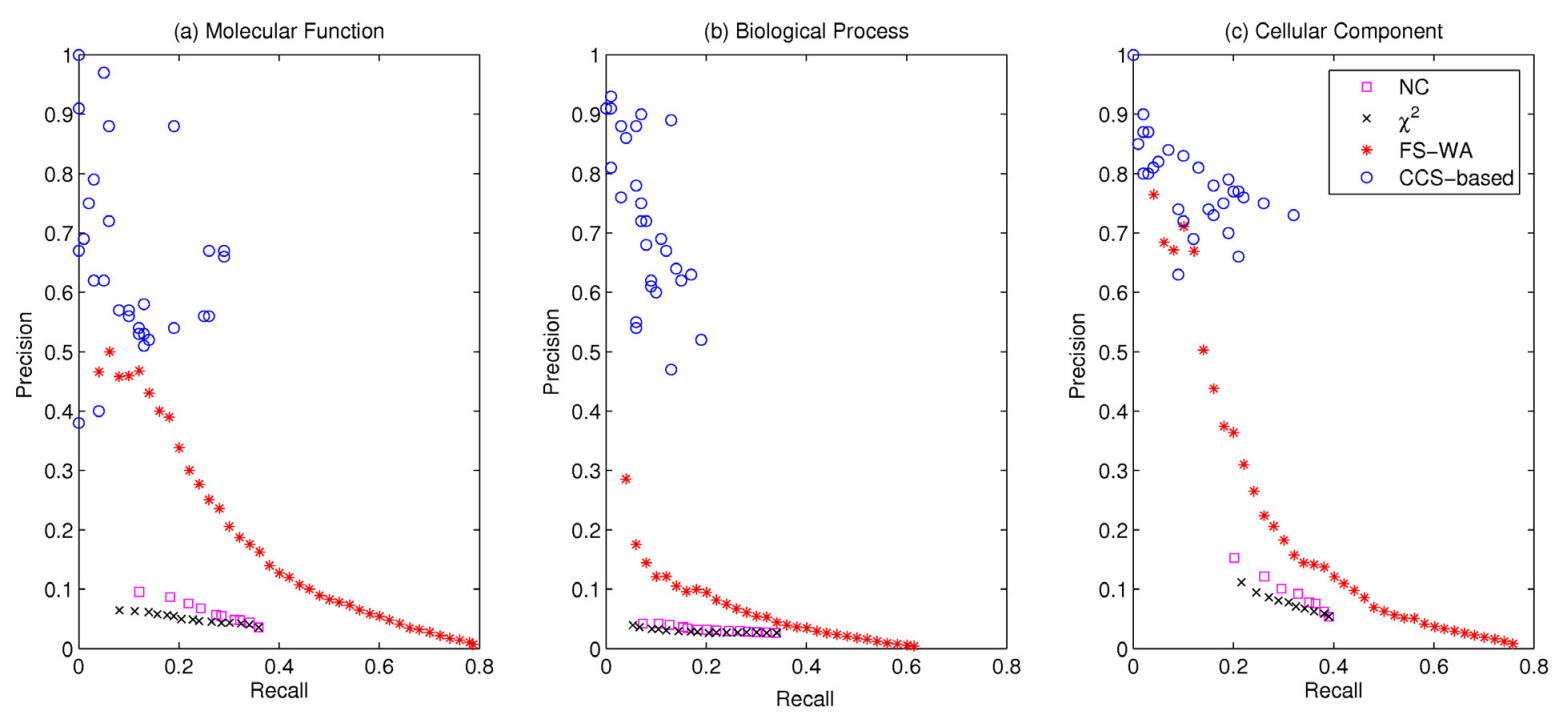
**Direct performance comparison for human**. Comparing precision and recall of function predictions for proteins involved in CCS from weighted average (WA), neighbor counting (NC), χ^2 ^statistics and CCS-based approach for (a) molecular function, (b) biological process and (c) cellular component. CCS-based results are retrieved from different similarity thresholds and species combinations.

We compare the performance of our CCS-based prediction approach against *Neighbor Counting *(NC) [19], χ^2 ^statistics [16] and *FS-Weighted Averaging *(FS-WA) [27] considering only proteins that are involved in CCS. The performance of the individual methods on the complete data is shown in Additional File 1, Figure S6. Figure 4 presents precision - recall graphs (based on varying thresholds) for predictions for human proteins separated by the three GO subontologies. CCS-based function prediction significantly outperforms *NC *and χ^2 ^statistics. Precision and recall obtained from the latter two are very low and even below our baselines. This also holds for yeast and fly (see Additional File 1, Figure S7 and S8).

When comparing *FS-WA *results with our approach, CCS-based function prediction performs consistently as well or better. Depending on species and subontology we achieve either higher precision at a similar recall or an improved precision and recall. Especially, when considering molecular function and biological process in human (see Figure 4) our method clearly outperforms *FS-WA*.

## Discussion

We presented a novel approach to predict protein functions that uses data from multiple species and combines three different sources of evidences for functional similarity: Orthology relationships, evolutionary conservation of functional modules in protein networks, and direct and indirect protein-protein interactions. Integrating these evidences into a single prediction algorithm overcomes the individual weaknesses of the base methods: (1) Orthology restricts prediction to proteins that have at least one orthologous protein with known function and exhibits a very low precision. (2) Considering only protein-protein interactions disregards the power of comparative genomics, leading to low coverage in organisms where PPI data is not available in abundance. (3) Using only functional modules within protein networks yields high precision, but strongly affects recall on a species basis, as only highly conserved functions performed by dense protein clusters can be predicted. We showed that combining these methods leads to high precision predictions with very good coverage. Essentially, we achieve high precision by looking only at subgraphs conserved in multiple species without restricting them to dense modules. Furthermore, we achieve high coverage when considering multiple species, by using a relaxed definition of interologs, and by transferring function from PPI neighbors and from orthologous proteins. Altogether, our method predicts thousands of protein functions for every species included in the analysis at varying, yet always high levels of precision (see Table 5).

**Table 5:** Overall function prediction statistics across all species combinations.

species	# terms	P	**R**_***pp***_	# terms	P	**R**_***pp***_	# terms	P	**R**_***pp***_
*rno*	17430	0.50	0.12	9110	0.57	0.14	5738	0.75	0.20
*mmu*	26089	0.47	0.13	11441	0.59	0.16	5246	0.81	0.23
*hsa*	62833	0.44	0.14	22911	0.59	0.17	12317	0.76	0.39
*dme*	26155	0.56	0.19	19463	0.60	0.20	10455	0.75	0.30
*cel*	4098	0.52	0.14	1983	0.60	0.18	1332	0.71	0.24
*sce*	10666	0.66	0.23	9013	0.69	0.25	6335	0.81	0.35

Total number of newly derived GO annotations and their estimated average precision (P) and per-protein recall (Rpp) for each species using low, medium and high similarity thresholds.

### Network Comparison

For comparing protein interaction networks we used two definitions for determining interologs: the strict and the relaxed definition when studying either two or more than two species, respectively. We also experimented with using the strict interolog definition for multiple species, but this often results in zero or only very few and small CCS within species groups (see Additional File 1, Table S2 for strict vs. relaxed results). This leads to a small but highly precise set of function predictions. Being less strict leads to a significant improvement in the coverage of our prediction method at comparable precision (see Additional File 1, Section S2.1 and Table S9). In turn, we also tested the effect of applying a relaxed definition of interologs to species pairs. This leads to very few (often only one) yet very large networks, as it only creates the union of interactions between orthologous proteins of the two species. However, this does not reflect evolutionary conservation of PPIs and therefore misses the important signals of functional conservation.

### Function Prediction

We evaluated or method in several ways using precision and recall, two baselines and three other function prediction methods. However, besides pure precision and recall values, an important property of any function prediction method is the specificity of its predicted terms. Clearly, predicting only very general terms is much simpler but much less useful than predicting terms close to the leaves of GO. Our methods predicts terms at a median level of 10 for cellular component, 8 for biological process, and 6 for molecular function. Thus, our method is capable of predicting quite specific functions (also see discussion of novel functions below).

Compared to other methods presented in the literature, our method has also the important property that it is not limited to so-called "informative" GO terms [64]. Many prediction methods use only GO terms that are associated to more than ten or 30 genes [26,27,62]. Such an approach implicitly disregards more specific annotations, although those are the most valuable ones. For example, in 2007 82.5% of GO annotations in human were associated to less than ten genes [65] leaving only 17.5% as annotation basis. GO-based methods have been shown to result in higher precisions when applied on a small number of frequently annotated GO terms. In contrast, we are able to generate accurate predictions also for rarely used GO terms.

#### Comparison to Baselines

Compared to the orthology baseline, including CCS yields precisions up to 10-times higher, confirming that information on conserved interactions is a very effective filter for avoiding false positive predictions across orthology relationships. Compared to the neighbor baseline, considering CCS also leads to a clear and significant increase in precision. This effect can be explained by the fact that using interologs (strict or relaxed) instead of single interactions largely improves reliability of PPI data [66], since false positive PPIs are unlikely to be reproduced across multiple species.

Our results show that combining various evidences into a single and comprehensive method leads to improved results. Evidently, the predictions made by different methods using information from different species complement each other quite well instead of only predicting the same functions again and again. However, the concrete approach has to be chosen with care. We showed that good results can only be achieved when using a proper definition of interaction conservation and when treating large CCS in an adequate manner. Failing to do so either restricts coverage of the method or leads to a higher false positive rates.

#### Comparison to other Function Prediction Methods

We compared precision and recall of our approach to Neighbor Counting, χ^2 ^statistics and FS-Weighted Averaging (see Methods). Our combined CCS-based approach significantly outperforms Neighbor Counting and χ^2 ^statistics, especially in terms of precision. Moreover, we perform comparably well or better against FS-Weighted Averaging, mostly achieving much higher precision at higher recall. Notably, our method achieves better results especially for species without comprehensive PPI coverage, such as human. However, precision for Neighbor Counting and χ^2 ^is significantly lower (on the entire data sets, see Additional File 1, Figure S6, and the filtered protein sets) than reported in the respective original publications [16,19]. There are three explanations for this drop (from ~70% to 15% precision). First, both methods originally were evaluated only on the functional classification scheme from YPD. This scheme covers, similar to GO, three categories of yeast protein function: biochemical function, cellular role and subcellular localization. However, categories have only 57, 41 and 22 members, respectively. Compared to our evaluation using GO, in which methods have to chose between up-to 17398 functional categories, this increases the chances to predict correct terms purely by chance. Furthermore, yeast is a particularly well-studied organism, while we applied the method also to less-well covered species. A similar performance drop was observed by Chua *et al*. [62], which also applied both methods to GO term prediction, with precision decreasing to 60% (*NC*) and 20% (χ^2^) for yeast and 20% (*NC*) and 16% (χ^2^) for fly. The second point concerns the amount of interaction data. For example, results from [19] are based on only 2,709 interactions among 2,039 proteins. In contrast, we integrated six different databases, leading to, for instance, almost 70,000 interactions for 6,500 proteins in yeast. Thus, we cover many more proteins and interactions which also increases the probability of false positives. Third, many prediction methods, including the two studies compared to here, consider only annotated proteins with at least one annotated interaction partner for their studies [16,19,26,27,62]. We did not exclude those proteins because we believe that especially weakly or un-annotated proteins must be a primary target for function prediction. In our combined approach, such proteins often receive functions from orthologous proteins in other species, an option missing in Neighbor Counting and χ^2^. However, functions predicted for non-annotated proteins are necessarily counted as false positives although these are truly novel findings. Thus, disregarding such proteins results in higher precisions.

We did not compare to purely module-based prediction methods, as link-based techniques have been shown to outperform those [3,37]. However, we evaluated the effect of requiring CCS to be "module-like", i.e., to exhibit a certain density of interactions between its members. CCS-density is defined as $\frac{2*|E|}{|V|\left(|V|-1\right)}$ where *E *presents the edges and *V *denotes the nodes within a CCS. As expected, filtering CCS according to their density considerably improves precision (see Additional File 1, Figure S9), e.g. in fly from 80% without filtering to 90% for a density of 0.7 and 95% for a density of 1, but this increase is at the cost of much fewer predictions (see Additional File 1, Section S2.2 for a detailed discussion).

#### Effects of Size of Data Sets

**Figure 5:**
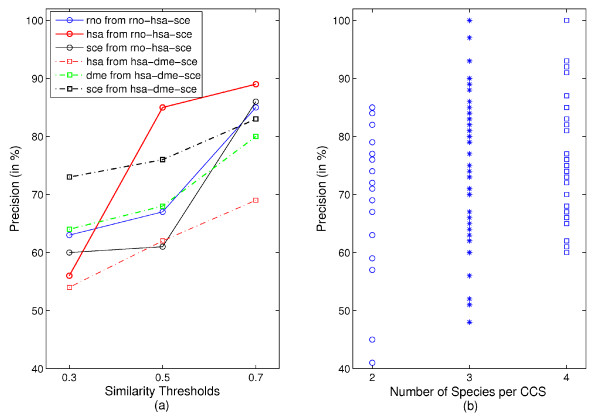
**Correlation of the prediction precision with (a) functional and (b) evolutionary conservation, respectively**. (a) Species-specific precision values for predictions derived from CCS among *rno-hsa-sce *(solid lines) and *hsa-dme-sce *(dashed lines). For each species we plot the estimated precision against the applied similarity threshold (low: 0.3, medium: 0.5, high: 0.7) that indicates the level of functional conservation. (b) Species-specific precision grouped by evolutionary conservation that is given by the increasing number of species involved in a CCS.

Results of our prediction method vary depending on the level of available annotations and PPIs for the species that are compared (see Additional File 1, Section S3.1 for a discussion of the data). They are better when well-studied species, such as yeast or fly, are involved. This is an inherent property of methods that transfer annotations, since better annotated species provide more source functions. This property underpins the importance of comparative genomics for elucidating the function of human proteins. It is also clearly visible that prediction precision is correlated to the threshold for functional conservation (see Figure 5a) and increases with the degree of evolutionary conservation of a CCS - from pairwise to multiple network comparisons (Figure 5b). Obviously, the functional conservation threshold is an important possibility to tune or method to the specific needs of an application. The higher the functional conservation, the higher is the precision of the predictions.

Note that in any gold standard evaluation as ours, new findings are always counted as false positives, independently of their real, biological truthfulness. Consequently, prediction methods perform better on well-studied organisms than on species that are functionally less well characterized. The precision values we report therefore should be considered as lower bounds on the true precision.

#### Performance on Weakly and Non-Annotated Proteins

An important goal of protein function prediction is to derive novel functions for proteins without any or with only very little functional information. Thus, we analyzed how our method performs on such proteins. We define as a weakly annotated protein (WAP) any protein which has at most two terms assigned a-priori in our data. For WAP, we count annotations as new if they are more specific than the existing ones or if they belong to another sub-branch in the subontology. Note that such annotations are counted as false positives in our evaluation as they cannot be validated from our gold standard data.

**Figure 6:**
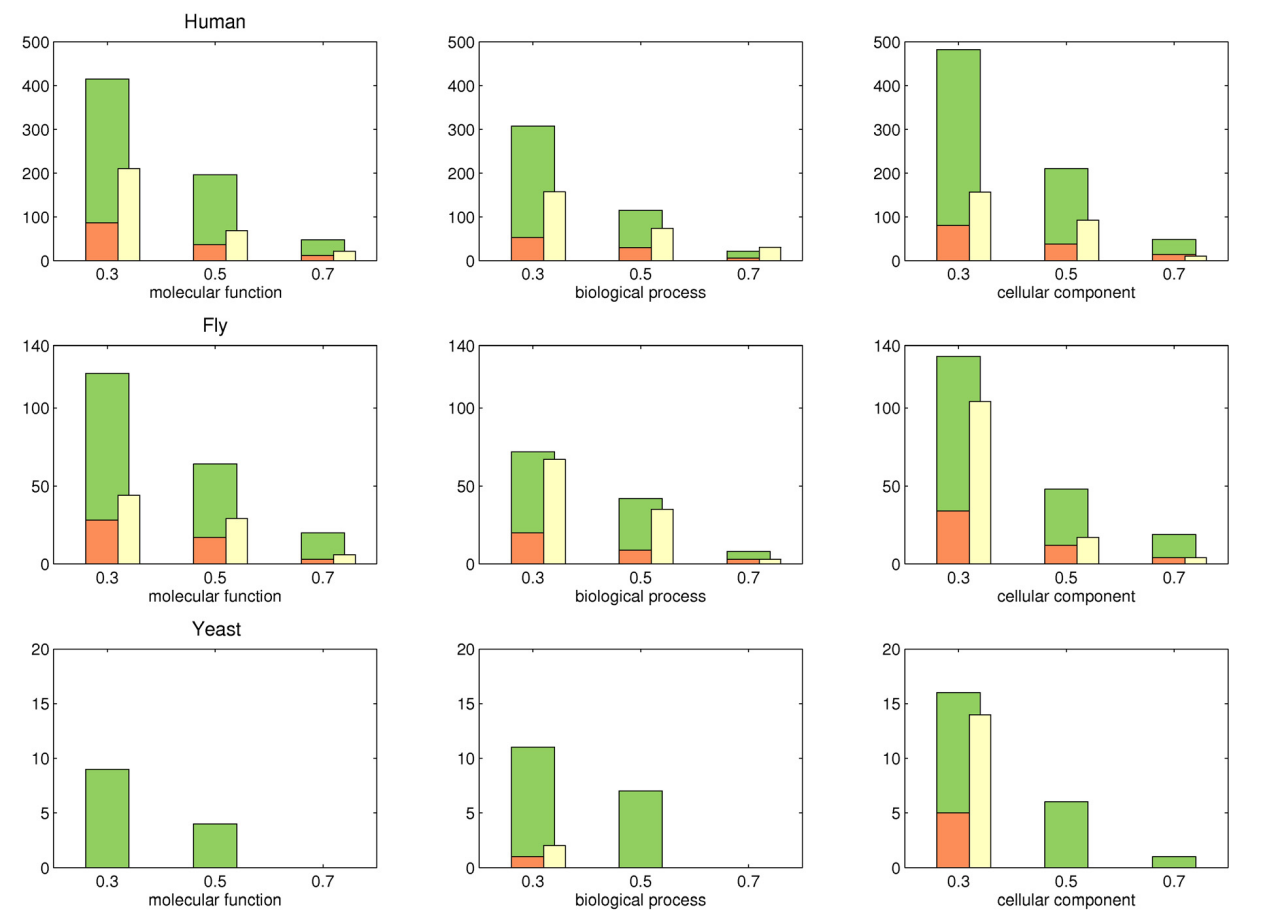
**Number of predicted functions for proteins without annotations within CCS from hsa-dme-sce**. For each subontology and similarity threshold the number of proteins without any annotation (olive), the number of proteins that receive new annotations (orange) and the total number of novel annotations are shown (yellow). Recall that a higher coherence threshold for CCS leads to less proteins being included in function predictions; thus, numbers generally decrease with higher thresholds.

Results from comparing *hsa-dme-sce *are shown in Figure 6 and Additional File 1, Figure S10. As expected, the highest number of proteins without any annotation can be found in human. Annotation coverage of fly is not as good as for yeast but still much better than in human. For example, CCS at threshold 0.3 contain ~300 human proteins without any functional annotation in biological process. By means of our method, we predict 156 annotation for 52 of those proteins. Similarly, 20 fly proteins out of 72 are annotated with 67 GO annotation in biological process. But also well-studied species still contain many WAPs and benefit from our approach. For instance, about 380 yeast proteins are only weakly characterized for cellular component and for more than a quarter of them we predict about 200 functions. Note that the fraction of WAPs receiving new annotations decreases with the increase of the similarity threshold for each species.

### Predictions for Selected Human Proteins

In the following, we discuss specific predictions for proteins that are relevant for colorectal cancer. Note that these predictions were counted as false positives in our evaluation because they are not contained in the Gene Ontology annotations at all or only marked as putative (mostly "inferred from electronic annotation", IEA). However, we show that many predictions already have strong experimental support in the literature. Thus, the group of novel predictions falls into two classes - those that, given the current literature, can be considered as true but have not yet made it into the annotation databases and those for which we could not find conclusive evidence in the literature. We argue that, given the large amount of predictions that fall in the first class, predictions from the second class should be considered as promising candidates for further studies.

We discuss predicted functions for the gene products of *MLH1*, *PMS2 *and *EPHB4*, all of which have an established importance for colorectal cancer [67,68]. Overall, literature curation largely confirms the predictions for these three genes by different experimental studies.

#### MLH1 and PMS2

**Figure 7:**
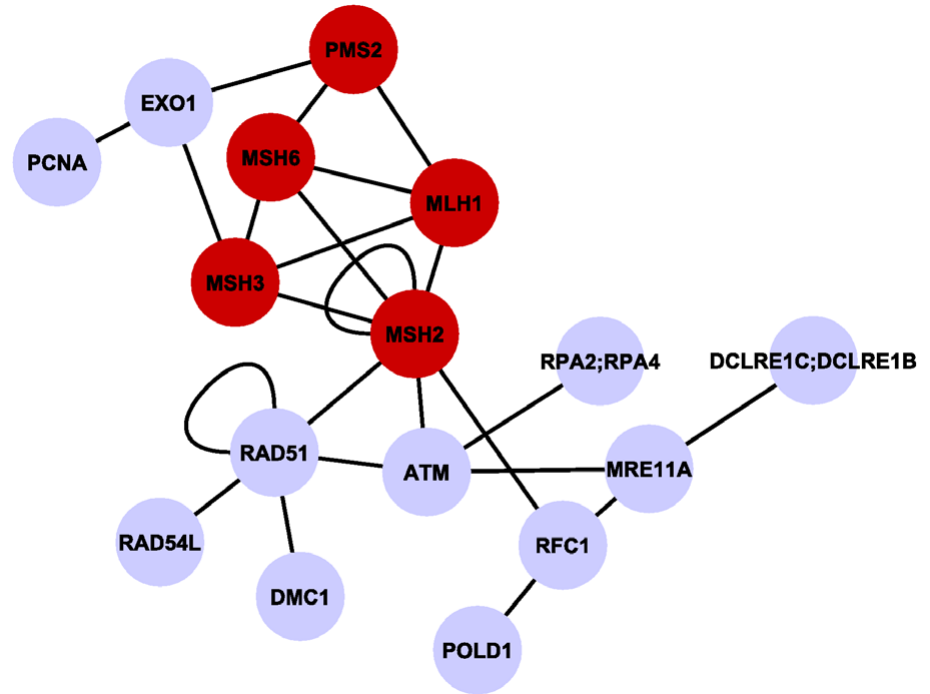
**Components of the post-replicative DNA mismatch repair system (MMR)**. CCS derived from PPI network comparison between human and yeast. Subgraph clusters proteins that are involved in mismatch repair (protein names correspond to human proteins). Proteins associated to colorectal cancer are indicated in red.

The DNA mismatch repair protein MLH1 and the mismatch repair endonuclease PMS2 belong to the main components of the post-replicative DNA mismatch repair (MMR) system (see Figure 7) [69]. The MMR system is required for correcting base mismatches and insertion or deletion loops resulting from DNA replication, DNA damage, or from recombination events between non-identical sequences during meiosis [70]. Curated annotation for *MLH1 *and *PMS2 *from UniProt and EntrezGene and newly inferred functions are listed in Additional File 1, Table S10 and S11.

The majority of our predictions (terms are set *italics *in the following) is directly related to the functionality of the MutLα complex which is formed by *MLH1 *and *PMS2*. Rich supporting evidence can be found from the respective orthologs in yeast and mouse. For instance, *PMS1*, the *PMS2 *ortholog in yeast, contributes to *dinucleotide insertion or deletion binding*, *loop DNA binding *[71]. *Mlh1*, the mouse ortholog of *MLH1*, is annotated to *guanine/thymine mispair binding *[72] and likely plays a role in the formation, stabilization and/or the resolution of Holliday junction intermediates (*four-way junction DNA binding*) [73]. High and low affinity ATP binding sites have been observed for *MLH1 *and *PMS1 *in yeast [74] which supports the *ATP binding *and *ATPase activity *predictions for their human orthologs [75]. Moreover, *PMS2 *contains a conserved metal-binding motif that constitutes part of the active site for the endonuclease activity of the protein and might enable *magnesium ion binding *[76]. Considering *protein homodimerization activity*, the dysregulated gene expression of *PMS2*, either as a monomer or homodimer, can disrupt MMR function in mammalian cells [77]. Note that, although we support our predictions by literature evidences that are mostly based on orthologs, our algorithm actually inferred them from conserved interaction partners as the orthologs in most cases do not carry the annotation we found in the literature.

Our algorithm also generates a number of predictions that are not as clearly supported by the existing literature, such as *guanine/thymine mispair binding*, *single guanine *and *thymine insertion binding *or *oxidized DNA binding*. Moreover, we associate both proteins to *base-excision repair *as well as *postreplication repair *and *MLH1 *to *maintenance of DNA repeat elements*. These are interesting hypotheses supported by recent findings from Erdeniz *et al*. who suggested that the endonuclease activity of *PMS2 *in MutLα is not only important in MMR-dependent mutation avoidance but also for suppression of homologous recombination, DNA damage signaling, and damage response functions [78]. Association of yeast *PMS1 *with *meiotic mismatch repair *and *DNA recombination *[79] further support these predictions. Regarding their cellular components both proteins are associated to the *MutLα complex *[67], an annotation predicted jointly from orthology and the CCS neighborhood. *MutLα complex *is a clearly sensible refinement of the existing annotation *nucleus *and only seven others genes are annotated to this term, which emphasizes the specificity of our method.

#### EPHB4

Ephrin type-B receptor 4 is a transmembrane receptor for the ephrin-B family. It belongs to the family of receptor tyrosine kinase (RTK) and is usually expressed in endothelial and neuronal cells. Known and predicted functional annotations are displayed in Additional File 1, Table S12.

Several predicted functions, such as *protein*, *enzyme *and *ATP binding*, *SH3/SH2 adaptor *and *enzyme regulator activity *and *protein amino acid phosphorylation*, derived both from conserved interactions and orthology, are evidently consistent with the characteristics of receptor tyrosine kinases.

Two functions inferred by orthology are *transmembrane-ephrin receptor activity *and *transmembrane receptor protein tyrosine kinase signaling pathways*. Both are supported by annotations from highly related receptors, such as *Ephb1 *in mouse and *EPHB2 *in human [80,81]. Less evident predictions are, for instance, *cell-cell signaling *[82], *cell migration *[83], *angiogenesis *and *behavior *[84]. These functions were not predicted by orthology alone but only in combination with the conserved interaction neighborhood of *EPHB4*. *EPHB4 *participates in the axon guidance pathway and in this context predictions like *axon guidance *or *axon guidance receptor activity *can be integrated [85-87].

## Conclusion

Elucidating protein function is a major challenge in the post-genomic era. We developed a method for predicting protein function based on the structural and functional conservation of PPI subnetworks in multiple species. Our approach integrates three different sources of evidences for inferring functional similarity. Altogether, we employ orthology relationships, evolutionary conservation of functional modules in protein networks, and direct and indirect protein-protein interactions for deriving novel functions for uncharacterized proteins. Using our method we derive thousands of protein functions for every species in our study at varying, yet high levels of precision. Thus, combining orthology relationships, functional modules and PPI neighborhood into a single, comprehensive prediction method yields high-quality predictions with very good coverage. In comparison against three other function prediction approaches, Neighbor Counting, ^2 ^statistics, and FS-Weighted Averaging, our CCS-based prediction strategy performs comparably well or significantly better, especially in terms of precision.

Additionally, we predict a large amount of novel functions for a number of poorly or non-annotated proteins that can not be validated directly. However, this shows that our method also generates novel functional knowledge rather than only reproducing known functions for well-characterized proteins. The manual curation of predictions for three selected proteins confirms their high quality and precision as many predictions already have strong experimental support in the literature.

Apart from the promising results of our prediction approach, our method currently only provides lists of yes/no predictions. This binary behavior is implicit in the way we compute CCS and how we determine predicted terms and targets of prediction. For further improvement and applicability we plan to derive confidence scores for each prediction based on the multiple biological evidences. Predictions ranked by reliability will provide a method of selection for focusing experimental resources on hypotheses (predictions) that are more likely to be true. This is essential for experimental biologists to decide which proteins and predictions should be investigated further, e.g. in follow-up experiments.

## Competing interests

The authors declare that they have no competing interests.

## Authors' contributions

SJ: developed the methods to identify conserved protein interaction subgraphs and to predict protein functions, carried out the studies described in this paper and contributed to the manuscript. CS: contributed to the manuscript. UL: conceived the study and contributed to the manuscript. All authors read and approved the final manuscript.

## Supplementary Material

Additional file 1 **Supplementary Material**. The Supplementary Material includes supplementary figures and tables as well as additional analysis. Click here for file

Additional file 2 **Complete results of the strict and relaxed network comparisons**. This files contains the complete results of the strict and relaxed network comparisons for pairs of species and three, four, five and six species combinations. The number of OrthoMCL groups, interologs from strict and relaxed definition as well as the total number of CCS and the size of the largest CCS are given. Click here for file

Additional file 3 **Complete results of the CCS-based function prediction approach**. This file contains the complete results of the combined CCS-based function prediction approach for pairs of species and three, four, five and six species combinations. CCS from strict and relaxed network comparison are used depending on the species combinations. Click here for file
