# Supplementary material for: Combining modularity, conservation, and interactions of proteins significantly increases precision and coverage of protein function prediction
Source: BMC Genomics. 2010 Dec 20;11:717. doi: 10.1186/1471-2164-11-717 (PMC3017542; doi:10.1186/1471-2164-11-717)
Supplement: Additional file 1 — Supplementary Material. The Supplementary Material includes supplementary figures and tables as well as additional analysis. [file 1471-2164-11-717-S1.PDF]

Supplementary Material

Combining modularity, conservation, and interactions of proteins significantly increases precision and coverage of protein function prediction

Samira Jaeger[1], Christine Sers[2], Ulf Leser[1]

[1]Knowledge Management in Bioinformatics, Humboldt-Universität zu Berlin, Germany
[2]Institute of Pathology, Molecular Tumorpathology, University Medicine Charite, Germany
*E-mail: {sjaeger}@informatik.hu-berlin.de

S1 Supplementary Methods

S1.1 Functional coherence of CCS

We assess the functional coherence of a CCS by analyzing the GO annotations of the proteins within each CCS. We use semantic similarity to first determine the similarity of two GO terms. This is extended to measure the functional similarity of two proteins annotated with several GO terms. Finally, we compute for each CCS its average functional similarity within a species (Sim_{neigh} – similarity between neighbors) and across the species (Sim_{ortho} – similarity between orthologs).

S1.1.1 Semantic similarity between GO terms

To compute the semantic similarity between two GO terms we use the approach proposed by Lin (Lin, 1998). Following Lin's definition, the information content of a GO term t is defined as follows:

$$IC(t) = -log\left(\frac{freq(t)}{freq(root)}\right), \quad \text{(S1)}$$

where the frequency of a term is defined as the number of times a term or any of its descendants occurs. Thus, less frequent terms and terms with few occurring descendants are considered more informative.

Based on this measure, the semantic similarity between two terms is defined as the ratio of the information content of their most informative common ancestor and the information contents of both concepts (Lin, 1998). The information content of the most informative common ancestor is given by:

$$shareIC(t_1, t_2) = max\left\{IC(t)|t \in CA(t_1, t_2)\right\}, \quad \text{(S2)}$$

where $CA(t_1, t_2)$ is the set of all common ancestors between terms t_1 and t_2. The similarity between two terms is then defined as:

$$sim(t_1, t_2) = \frac{2 * shareIC(t_1, t_2)}{IC(t_1) + IC(t_2)}. \quad \text{(S3)}$$

S1.1.2 Semantic similarity between proteins

The semantic similarity between proteins is determined based on the similarity of their associated GO terms. Since often proteins are annotated with more than one term, the similarity of a protein p to a group g of terms is defined as the average similarity of its terms to their most similar terms in g (Couto *et al.*, 2007) (where $t(p)$ is the set of terms annotated to protein p):

$$Sim(p, g) = \frac{\sum\limits_{t_1 \in t(p)} max\left\{sim(t_1, t_2)|t_2 \in g\right\}}{|t(p)|} \quad \text{(S4)}$$

We use the definition of Couto *et al.* (Couto *et al.*, 2007) to determine the GO similarity between two proteins which is defined as the average similarity of their GO terms:

$$GO_{Sim}(p_1, p_2) = \frac{Sim(p_1, t(p_2)) + Sim(p_2, t(p_1))}{2}. \quad \text{(S5)}$$

S1.1.3 Functional similarity within CCS

Finally, we determine the functional similarity within CCS using the concept for semantic similarity concepts between proteins as defined above. Given the two sources of conservation in CCS, we measure functional similarity separately between ortholgs across the species and between interacting proteins within a species. Each CCS is characterized by its set of V nodes (orthologous groups) and E edges (interologs), and the k species it has been identified in.

Orthology-based similarity – Sim_{ortho} For functional similarity between orthologs we first compute the pairwise similarities between the proteins within an orthologous group o. Subsequently, we add all pairwise protein similarities and divide the sum by the number n of protein comparisons within the group o ($n = \frac{k*(k-1)}{2}$) to obtain an average score for o:

$$GO_{Sim}(o) = \frac{\sum\limits_{i,j(i<j)}^{k} GO_{Sim}(p_i, p_j)}{n}. \quad \text{(S6)}$$

The individual similarity scores of each group are then added and divided by the number of orthologous groups (|V|) in the CCS:

$$Sim_{ortho}(\text{CCS}) = \frac{\sum\limits_{o \in \text{CCS}} GO_{Sim}(o)}{|\text{V}|}. \quad \text{(S7)}$$

Interactor-based similarity – Sim_{neigh} The similarity measure Sim_{neigh} determines the functional similarity between the interaction partners within a CCS. To compute Sim_{neigh} we determine the functional similarity between all interacting proteins of the same species x within the CCS. Pairwise similarities are then added and divided by the number of edges (|E|) in the CCS:

$$Sim_{neigh_x}(\text{CCS}) = \frac{\sum\limits_{(u,v) \in \text{E}, u<v} GO_{Sim}(p_u, p_v)}{|\text{E}|}. \quad \text{(S8)}$$

Depending on the similarity of the GO annotations of the proteins of a CCS, Sim_{ortho} and Sim_{neigh} range between 0 and 1, whereby 1 indicated functional equality and 0 indicates maximal functional distance. CCS lacking protein annotations result in a decreased semantic similarity due to missing annotations.

S1.2 Standardized z-score

Orthologous groups that differ significantly in their individual functional similarity ($GO_{Sim}(o)$) from the similarity score of the CCS (Sim_{ortho}) are determined by using the standardized z-score (Freedman *et al.*, 1998). The z-score specifies the difference between the similarity within an orthologous group and the similarity of the CCS normalized by the standard deviation of the orthologous similarites in the CCS (std_{ccs}):

$$z\text{-}score(o) = \frac{GO_{Sim}(o) - Sim_{ortho}(\text{CCS})}{\text{std}_{\text{ccs}}} \quad \text{(S9)}$$

On the basis of the z-score we derive a p-value to determine whether an observed difference is signficant. Differences with a p-value smaller than the significance level of $\alpha = 0.01$ are considered to be significant.

S1.3 Prediction using neighboring proteins

First, we calculate semantic similarities between all proteins interacting with the candidate protein c and consider all neighbors with a functional similarity above a threshold t as 'similar' neighbors.

$$similar\_neighbors(c) = \{p \in N(c) | GO_{Sim}(c, p) > t\} \tag{S10}$$

Next, we generate a set of potential annotations including all GO terms that are associated to at least one of the neighbors (where $GO(p)$ denotes the set of GO terms annotated to a protein p):

$$GO_{neighbors}(c) = \left\{ \bigcup_{p \in similar\_neighbors(c)} GO(p) \right\} \tag{S11}$$

Given the set of potential GO annotations and a candidate protein, we determine for each GO term g whether the similar neighbors are annotated to g or not.

$$annotated(c, g) = \{p \in similar\_neighbors(c) | g \in GO(p)\} \tag{S12}$$

If the fraction of 'similar' neighbors exceeds a threshold f, we predict the GO term to the candidate protein. This procedure is applied to all potential GO annotation to derive novel function for a candidate protein c:

$$prediction_{GO}(c) = \{g \in GO_{neighbors} | \, |annotated(c, g)| > f\} \, . \tag{S13}$$

S1.4 Adjustments for comparing CCS-based function prediction to other methods

For comparing *Neighbor Counting*, χ^2 *statistics* and *FS-Weighted Averaging* with our CCS-based approach we apply a script provided by Chua *et al.* that implements the three methods. This script was also used to perform the comparison of those methods in Chua *et al.* (2006, 2007). To allow for a fair comparison with the CCS-based prediction we use the script without limiting GO terms only to informative annotations and certain GO levels since we did not exclude them in our approach other than Chua *et al.* (2006, 2007). Moreover, for direct comparison we only consider proteins that are involved in CCS when evaluating *Neighbor Counting*, χ^2 *statistics* and *FS-Weighted Averaging* to generate comparable precision-recall graphs. On the other hand, our interaction data sets do not provide well-defined experimental sources. Therefore, interaction reliabilities can not be estimated and used within the weighted average method. However, when applying FS-Weighted Averaging to our data (default parameters), the results correlate with the outcomes shown in Chua *et al.* (2007). The individual performance of each method on the complete interaction data is shown in Figure S6.

S2 Supplementary Results

S2.1 Strict vs Relaxed CCS

Outcomes of strict and relaxed network comparisons are specified in Table S2.

The usage of the relaxed definition considerably increases the number of qualifying CCS for three or more species and utilizing relaxed CCS has a positive impact on function prediction. Table S9 compares the number of predicted terms along with precision and recall for CCS identified by strict and relaxed network comparisons among human, fly and yeast. Clearly, the number of predictions increases drastically (10- to 15-times), leading to a steep increase in prediction coverage. The influence on prediction precision is mostly smaller and in some cases negative. Notably, especially the predictions with highest reliability (threshold 0.7) are the least affected in terms of precision and often there is an increase, e.g. from 70% to 80% and 78% to 83% for *dme* and *sce*, respectively.

S2.2 Module density

Our method considers evolutionary conserved subgraphs with a high functional coherence as functional modules. However, the definition of functional modules differs from the traditional ones that primarily consider dense complexes, with a high connectivity or clustering coefficient, as modules (Bader and Hogue, 2003; Spirin and Mirny, 2003; Altaf-Ul-Amin *et al.*, 2006). To study the impact of subgraph density on function prediction we performed an experiment where we only consider candidate CCS with a certain density. CCS-density D is defined as:

$$D = \frac{2 * |E|}{|V|(|V| - 1)} \tag{S14}$$

where E presents the edges and V denotes the nodes within a CCS. The influence of high density on the number of candidate CCS and on the prediction precision is shown in Figure S9 for *hsa-dme-sce*. The number of candidate CCS decreases with an increasing density threshold, e.g. only 26 out of 177 CCS have a density above 0.7. On the other hand, the increasing density correlates with an increasing prediction precision at the expense of coverage. Figure S9 shows that limiting CCS to highly connected subgraphs increases prediction precision further, e.g. in fly from 80% without filtering to 90% for a density of 0.7 up to 95% for a density of 1.0. However, filtering for highly connected CCS disregards pathways and modules that are less linked, most likely due to the incompleteness of the data.

S3 Supplementary Discussion

S3.1 Data

The protein-protein interaction networks for each species are created by integrating PPI data from DIP, BIND, IntAct, BioGrid, MIPS–MPPI, MINT and HPRD. Proteins within the networks are additionally associated with sequences, protein domains and GO annotations. Protein sequences and domains are obtained from UniProtKb/SwissProt. GO annotations are retrieved from UniProtKb/Swiss-Prot, EntrezGene and species-specific databases, such as FlyBase, MGD, RGD, SGD and WormBase (see Table S1 for a detailed resource listing).

The different species-specific PPI networks vary significantly in their number of proteins and protein interaction as well as the median number of GO terms per proteins (see Table 1). The largest set of interactions is obtained for yeast followed by fly (in terms of the number of PPI per protein). Yeast and fly are both established model organisms that have been subject of several high-throughput experiments for large-scale protein interaction mapping (Uetz *et al.*, 2000; Walhout and Vidal, 2001; Gavin *et al.*, 2002; Giot *et al.*, 2003). The smallest set of interactions is derived for rat and mouse. The majority of interactions for both species has been obtained from the MIPS–MPPI database that focuses on (manually curated) mammalian protein-protein interaction data. Both species have been analyzed primarily in small-scale and hypothesis-driven studies and no large-scale analysis have been performed yet. On this account only few interaction data are available yet. The first interaction analysis in human have been small-scale studies for analyzing signal transduction pathways or disease. However, given the potential of protein interaction for elucidating function and disease mechanism, more and more large-scale studies have been performed for human and the variety of small-scale experiments and high-throughput studies contributed to an increasing number of interactions for human (Lehner and Fraser, 2004; Rual *et al.*, 2005; Stelzl *et al.*, 2005).

Considering the functional coverage of proteins within the PPI data sets, proteins of rat and mouse are functionally well-characterized. Proteins of both species are involved in protein interactions that have been detected in specific small-studies. These studies are often hypothesis-driven, e.g. knock-out-studies, and proteins are often analyzed in detail. Several supplementary information regarding for instance function, interaction partners and implications for diseases are often obtained for each protein from these studies. Thus, proteins of small-scale studies are often functionally better characterized than proteins from high-throughput studies. Although yeast and fly data are primarily derived from high-throughput studies, their proteins have fairly high functional coverage, mainly due to their role as model organisms in many research areas. In contrast to the other species, human and worm proteins are not well covered with functional annotations. Especially, for human functional elucidation of the whole genome is difficult.

S3.2 Evaluation criterion

For assessing the performance of our function prediction method we use leave-one cross-validation. We 'hide' selected annotations before applying our algorithm. Predicted terms are then compared to the held out annotations. When evaluating the correctness of a prediction we also consider the hierarchical structure of the Gene Ontology. Considering a prediction only to be correct if the protein is exactly annotated with this particular term is too strict since terms that are a little more general or more specific can equally contribute to a biological annotation. Thus, we count a GO term as correctly predicted if the proposed term was an ancestor of the original term or the term itself (in line with (Sharan *et al.*, 2005)).

To determine whether predictions are validated by direct ancestors of the original term or very generic annotations, we analyzed the distance within the GO graph between true positive prediction and the original annotation. Figure S2 shows the cumulative distances for true predictions inferred from CCS of *hsa-dme-sce*. The figure shows that the majority of predictions is confirmed by direct ancestors of the original term. Only 25% of the predictions have a distance of more than two levels to their original annotation. Predictions are in average only slightly more general (1.5 levels in GO) than the original annotations.

S4 Supplementary Figures

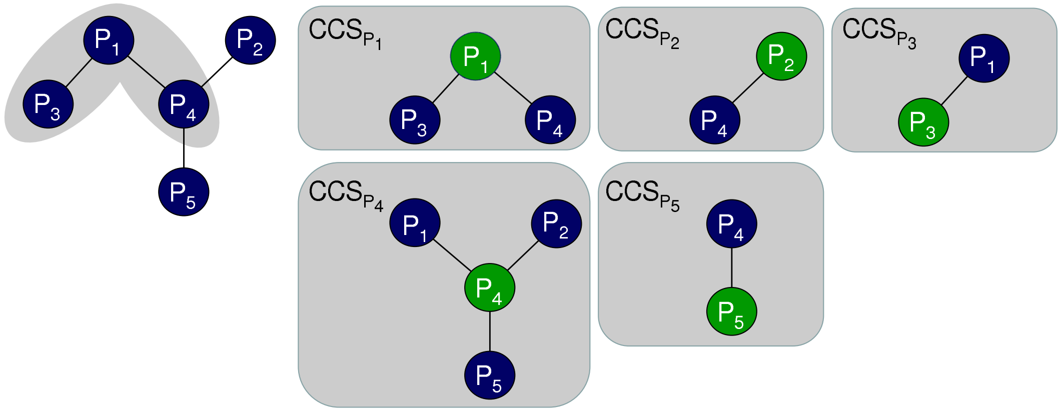

Figure S1. Illustration of the processing of large CCS. Large CCS are often heterogeneous since they encompass various functions. To employ large CCS for function prediction, we split CCS with more than 25 proteins into smaller, overlapping sub-subgraphs. Sub-subgraphs are built by considering each protein of the CCS as seed of a new, smaller CCS. All direct neighbors of this seed are added to the new CCS. Sub-subgraphs with less than three proteins are removed. For example, P_1 is used as seed and its direct neighbors P_3 and P_4 are added to form the new sub-subgraph CCS$_{P_1}$. Splitting the entire CCS results in five independent sub-subgraphs but only CCS_{P_1} and CCS_{P_4} are considered further for function prediction, as the rest is pruned (less than three proteins).

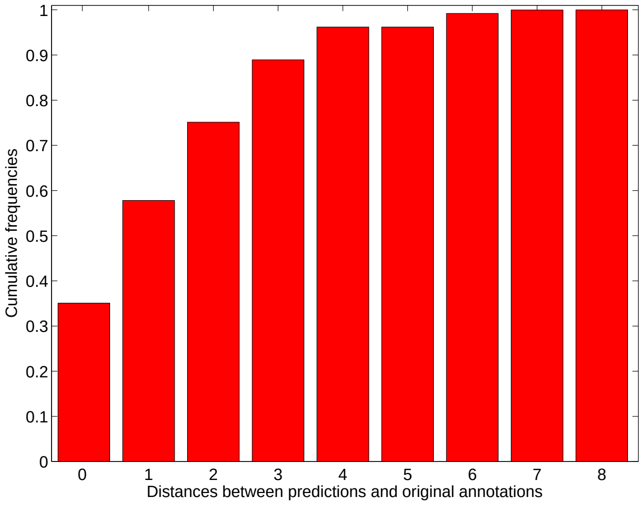

Figure S2. Cumulative distances between predictions and original annotations. Distances within the GO graph are determined between true positive predictions and the original annotation derived from CCS of *hsa*-*dme*-*sce* that show high functional similarities.

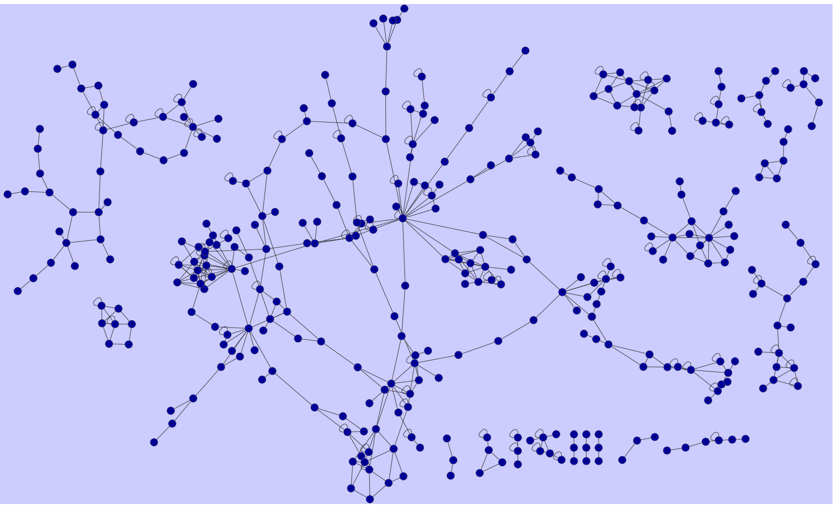

Figure S3. All 23 CCS with more than two proteins that are approximately conserved among human, fly, and yeast. The largest subgraph comprises 187 proteins and 316 interologs.

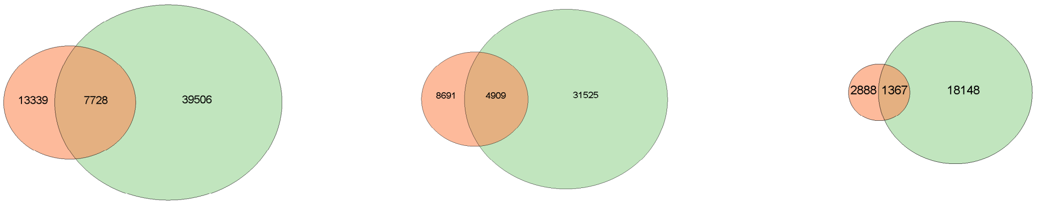

(a) Threshold 0.3 (b) Threshold 0.5 (c) Threshold 0.7

Figure S4. Overlap between predicted functions derived from the orthology- (orange) and link-based (olive) method for CCS from *hsa-dme-sce*.

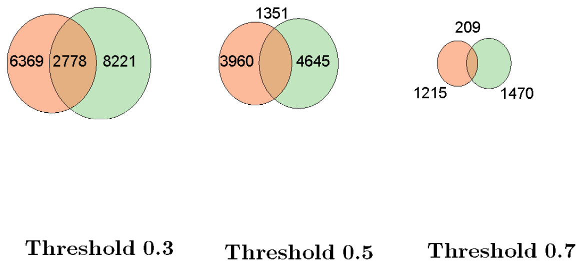

(a) Human

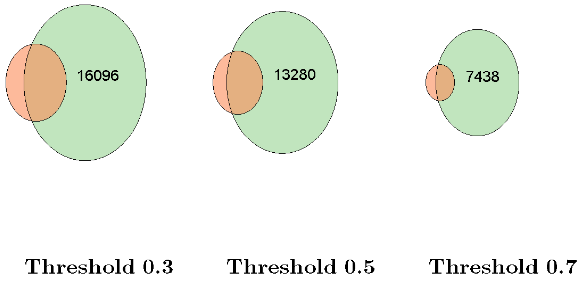

(b) Fly

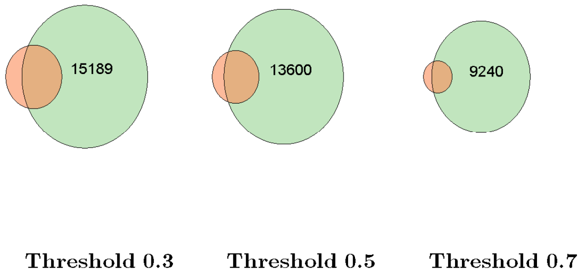

(c) Yeast

Figure S5. Overlap within predictions derived from the orthology- (orange) and link-based (olive) strategy for human, fly and yeast proteins from *hsa-dme-sce*.

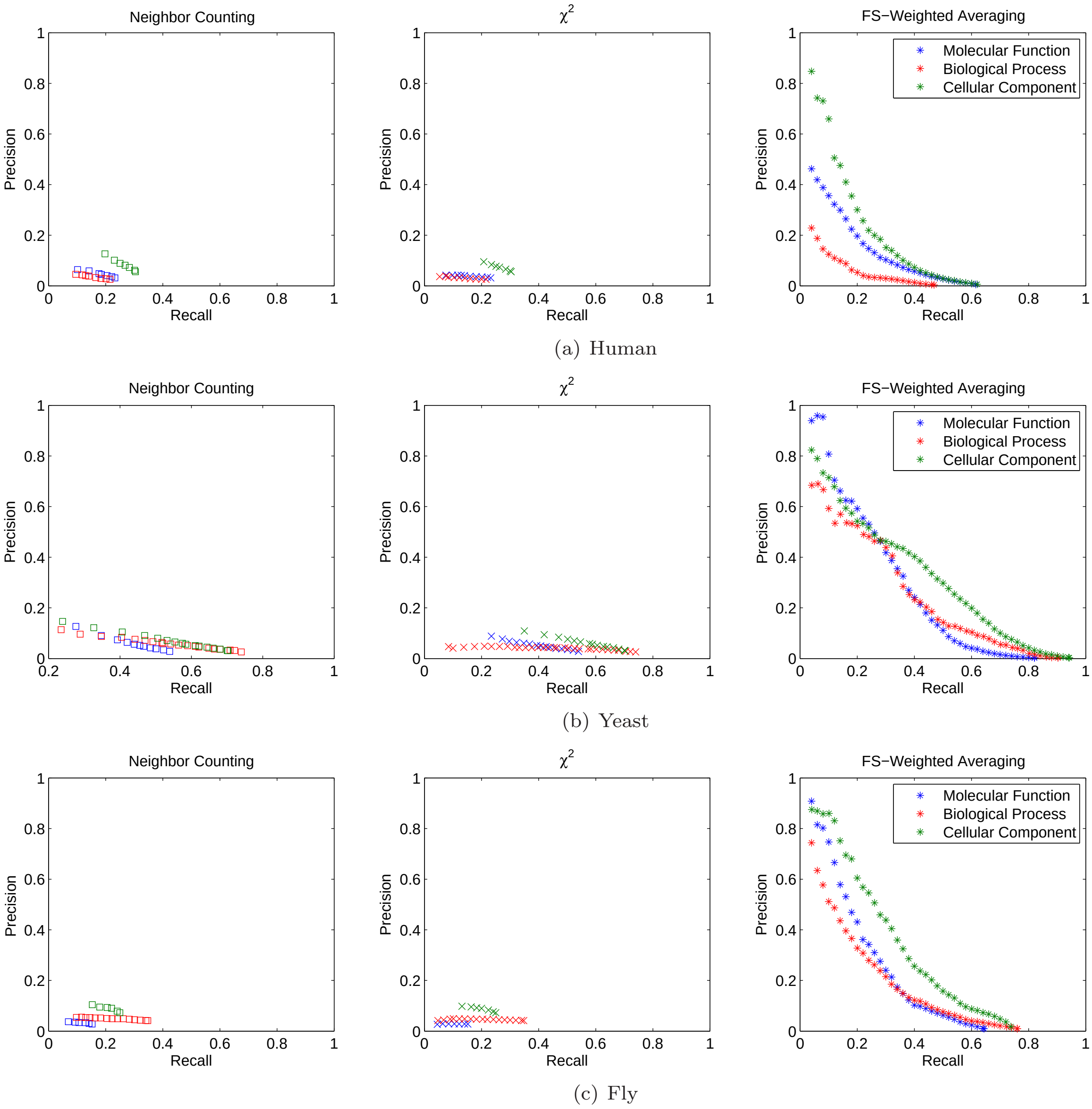

(a) Human

(b) Yeast

(c) Fly

Figure S6. Performance of the *Neighbor Counting*, χ^2 *statistics* and *FS-Weighted Averaging* on the complete interaction data of (a) human, (b) yeast and (c) fly for molecular function, biological process and cellular component.

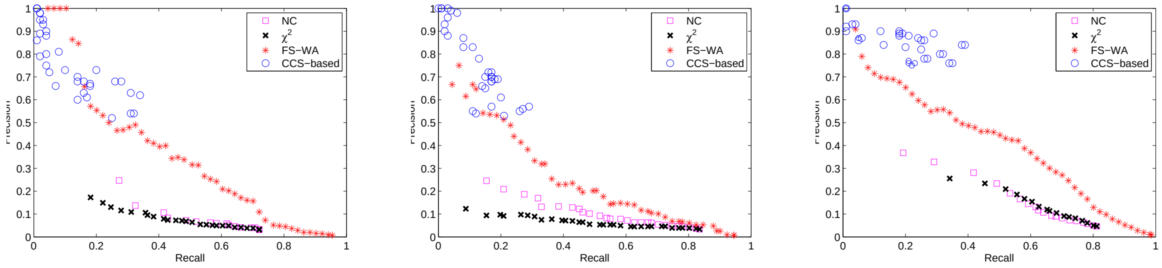

(a) Molecular function (b) Biological process (c) Cellular component

Figure S7. Direct performance comparison for yeast. Comparing precision and recall of function predictions for proteins involved in CCS from weighted average (FS-WA), neighbor counting (NC), χ^2 statistics and CCS-based approach for molecular function, biological process and cellular component. CCS-based results are retrieved from different similarity thresholds and species combinations.

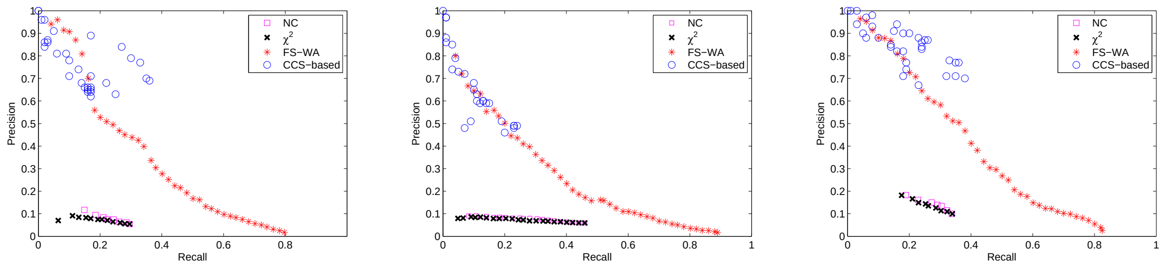

(a) Molecular function (b) Biological process (c) Cellular component

Figure S8. Direct performance comparison for fly. Comparing precision and recall of function predictions for proteins involved in CCS from weighted average (FS-WA), neighbor counting (NC), χ^2 statistics and CCS-based approach for molecular function, biological process and cellular component. CCS-based results are retrieved from different similarity thresholds and species combinations.

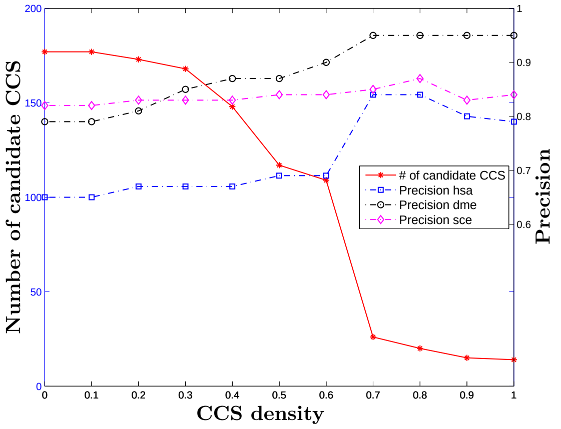

Figure S9. Impact of the CCS density on the number of qualifying CCS and the function prediction. The number of CCS for different densities is displayed (left y-axis) and the influence on the prediction precision (right y-axis).

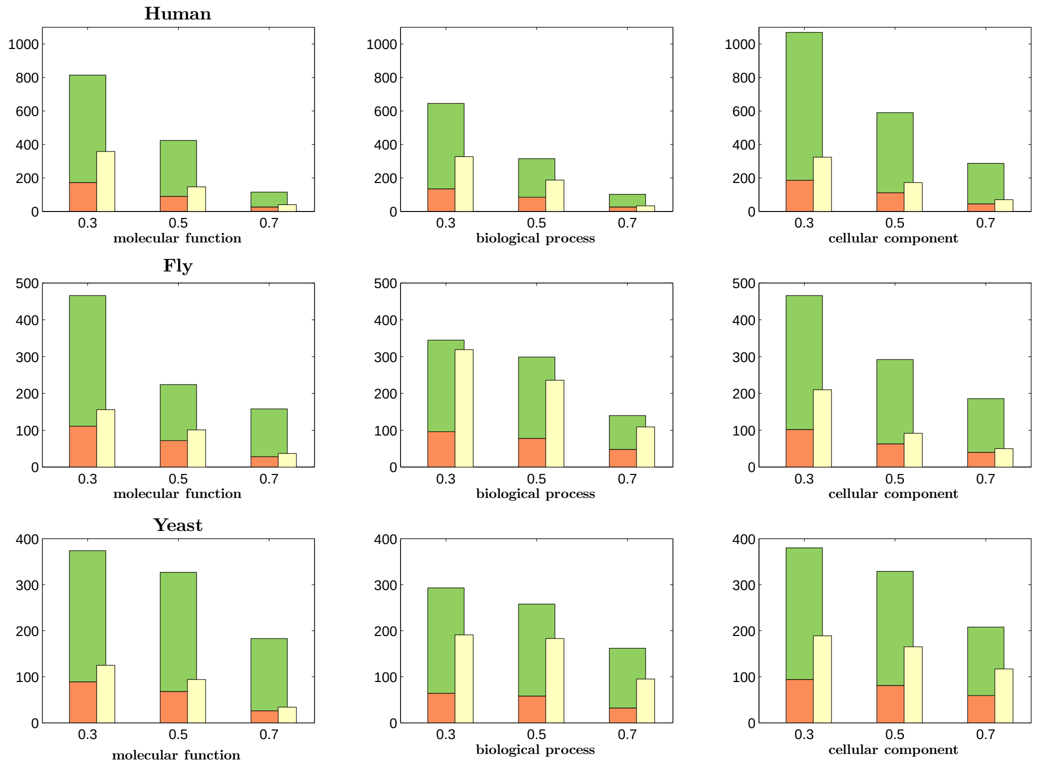

Figure S10. Function prediction for weakly annotated proteins (with less than three annotations) within CCS from *hsa-dme-sce*. For each subontology and similarity threshold the number of weakly annotated proteins (olive), the number of proteins that receive new annotations (orange) and the total number of novel annotations are shown (yellow). Note, only annotation that are more specific then existing ones are counted as novel.

S5 Supplementary Tables

Table S1. Data-specific resources and the number of retrieved data.

Resource	PPI Data	Protein Sequences	Protein domains	GO Annotation
DIP (Salwinski *et al.*, 2004)	21099	–	–	–
IntAct (Hermjakob *et al.*, 2004)	34765	–	–	–
BIND (Bader *et al.*, 2003)	23057	–	–	–
Mammalian MIPS (Pagel *et al.*, 2005)	454	–	–	–
HPRD (Peri *et al.*, 2003)	19310	–	–	–
MINT (Chatr-aryamontri *et al.*, 2007)	13763	–	–	–
BioGRID (Stark *et al.*, 2006)	92251	–	–	–
UniProtKb/Swiss-Prot (Boeckmann *et al.*, 2003)	–	35152	–	40324
EntrezGene (Wheeler *et al.*, 2008)	–	3930	–	15880
InterPro (Mulder and Apweiler, 2008)	–	–	29403	–
FlyBase (FlyBase Consortium, 2003)	–	–	–	23110
MGI (Bult *et al.*, 2008)	–	–	–	18241
RGD (Twigger *et al.*, 2007)	–	–	–	9911
SGD (Hong *et al.*, 2008)	–	–	–	25912
WormBase (Bieri *et al.*, 2007)	–	–	–	7248

Table S2. Complete results of the strict and relaxed network comparisons for pairs of species and three, four, five and six species combinations. The number of OrthoMCL groups, interologs from strict and relaxed definition as well as the total number of CCS and the size of the largest CCS are given.

Table S3. Complete results of the combined CCS-based prediction approach for pairs of species and three, four, five and six species combinations. CCS from strict and relaxed network comparison are used depending on the species combinations.

Table S4. Baseline for utilizing OrthoMCL orthology relationships for function prediction. Precision (P) and recall (R) are estimated from randomly sampling 1/3 of the OrthoMCL groups from a species combinations and predicting function along orthology across species within the groups. Results are averaged across 100 runs.

Species	# terms	∅ P (± std)	∅ R (± std)
dme	18665	0.06 (0.002)	0.40 (0.02)
sce	17057	0.06 (0.002)	0.29 (0.01)
rno	1021	0.11 (0.08)	0.23 (0.02)
hsa	1761	0.09 (0.07)	0.34 (0.03)
sce	1755	0.06 (0.006)	0.18 (0.03)
hsa	10971	0.08 (0.002)	0.28 (0.01)
dme	9280	0.07 (0.002)	0.27 (0.01)
sce	7752	0.07 (0.002)	0.16 (0.008)
hsa	951	0.10 (0.009)	0.06 (0.01)
dme	670	0.09 (0.01)	0.05 (0.006)
cel	1565	0.05 (0.01)	0.06 (0.006)
sce	616	0.07 (0.008)	0.03 (0.005)
mmu	1681	0.09 (0.006)	0.18 (0.02)
hsa	2056	0.08 (0.005)	0.17 (0.015)
dme	1791	0.07 (0.006)	0.11 (0.013)
sce	1499	0.06 (0.005)	0.07 (0.009)
∅	4943	0.08	0.23

Table S5. Baseline for link-based function prediction within species-specific PPI networks only without utilizing interologs. Precision (P) and recall (R) are estimated from sampling randomly 1/3 of the proteins of each interaction network independently of any species combination. Results are averaged across 100 runs.

Species	# terms	∅ P (± std)	∅ R (± std)
rno	3866	0.17 (0.02)	0.52 (0.04)
mmu	11367	0.15 (0.01)	0.61 (0.02)
hsa	80993	0.08 (0.002)	0.69 (0.008)
dme	108790	0.04 (0.001)	0.51 (0.01)
sce	175642	0.03 (0.001)	0.75 (0.006)
cel	7929	0.12 (0.01)	0.66 (0.02)
∅	64765	0.10	0.62

Table S6. Prediction results from exploiting only orthology relationships within CCS derived by exact (pairs) and approximative (multiple) network comparisons. Precision (P) and per-protein recall (R_{pp}) are estimated for low (0.3), medium (0.5) and high (0.7) functional similarity/conservation thresholds. Missing numbers indicate combinations where no CCS is homogeneously enough to pass the respective similarity threshold.

	0.3			0.5			0.7		
	# terms	P	R_{pp}	# terms	P	R_{pp}	# terms	P	R_{pp}
dme	3330	0.48	0.52	3075	0.47	0.56	884	0.63	0.80
sce	1604	0.59	0.53	1365	0.62	0.48	399	0.76	0.67
rno	293	0.84	0.26	51	0.96	0.31	8	1.0	0.57
hsa	620	0.56	0.33	98	1.0	0.37	16	1.0	0.76
sce	538	0.40	0.25	126	0.57	0.77	11	0.73	0.67
hsa	8071	0.43	0.51	2665	0.51	0.50	493	0.59	0.59
dme	4063	0.83	0.30	1526	0.78	0.34	372	0.75	0.52
sce	2907	0.88	0.30	1040	0.89	0.34	255	0.97	0.41
hsa	525	0.60	0.17	0	–	–	0	–	–
dme	216	0.76	0.08	0	–	–	0	–	–
cel	599	0.25	0.17	0	–	–	0	–	–
sce	160	0.84	0.07	0	–	–	0	–	–
mmu	735	0.67	0.44	216	0.85	0.57	0	–	–
hsa	844	0.55	0.36	332	0.61	0.55	0	–	–
dme	500	0.97	0.24	148	0.98	0.42	0	–	–
sce	411	0.97	0.25	154	0.98	0.42	0	–	–
∅	1589	0.66	0.30	900	0.77	0.47	305	0.80	0.62

Table S7. Precision (P) and per-protein recall (R_{pp}) for function prediction along interactions within CCS derived by exact (pairs) and approximative (multiple) network comparisons.

	0.3			0.5			0.7		
	# terms	P	R_{pp}	# terms	P	R_{pp}	# terms	P	R_{pp}
dme	3356	0.60	0.19	2212	0.67	0.25	739	0.84	0.28
sce	2511	0.64	0.20	1521	0.85	0.30	1031	0.87	0.40
rno	715	0.53	0.10	449	0.65	0.15	182	0.85	0.29
hsa	655	0.60	0.17	274	0.80	0.26	210	0.89	0.33
sce	1027	0.65	0.14	1016	0.65	0.14	230	0.87	0.19
hsa	8613	0.51	0.37	2507	0.65	0.50	453	0.75	0.50
dme	16678	0.60	0.36	15554	0.61	0.37	3371	0.72	0.47
sce	15364	0.72	0.41	15138	0.72	0.42	4124	0.84	0.55
hsa	2967	0.45	0.25	439	0.75	0.29	160	0.91	0.41
dme	5070	0.58	0.21	4586	0.59	0.23	866	0.81	0.29
cel	1891	0.56	0.26	1796	0.56	0.27	256	0.65	0.31
sce	5301	0.70	0.31	5126	0.71	0.32	1212	0.80	0.37
mmu	669	0.68	0.15	350	0.85	0.47	53	0.81	0.34
hsa	2640	0.47	0.27	1335	0.56	0.44	436	0.65	0.81
dme	5296	0.55	0.27	4583	0.57	0.30	1400	0.59	0.55
sce	4943	0.62	0.29	4875	0.62	0.31	2140	0.73	0.72
∅	4856	0.59	0.25	3860	0.68	0.31	1054	0.79	0.43

Table S8. Impact of splitting large CCS on precision (P) and per-protein recall (R_{pp}). CCS with more than 25 proteins are splitted into smaller sub-subgraphs before applying our function prediction method.

	0.3			0.5			0.7		
	# terms	P	R_{pp}	# terms	P	R_{pp}	# terms	P	R_{pp}
hsa	13347	0.54	0.18	8032	0.62	0.24	2778	0.69	0.25
dme	20531	0.64	0.19	16317	0.68	0.19	8708	0.80	0.27
sce	19400	0.73	0.20	17295	0.76	0.22	10644	0.83	0.28
hsa	3636	0.48	0.16	1317	0.69	0.23	505	0.72	0.21
dme	5539	0.60	0.15	4476	0.63	0.16	1641	0.83	0.23
cel	2687	0.50	0.18	1933	0.60	0.19	1042	0.67	0.21
sce	6523	0.70	0.19	5984	0.72	0.21	2568	0.85	0.27

Table S9. Impact of the strict and relaxed interolog definition on function prediction results of multiple species.

	0.3			0.5			0.7		
	# terms	P	R_{pp}	# terms	P	R_{pp}	# terms	P	R_{pp}
Strict									
hsa	167	0.58	0.24	15	0.80	0.06	0	-	-
dme	626	0.45	0.15	288	0.70	0.19	264	0.71	0.25
sce	276	0.73	0.16	264	0.73	0.17	134	0.78	0.32
Relaxed									
hsa	13347	0.54	0.18	8032	0.62	0.24	2778	0.69	0.25
dme	20531	0.64	0.19	16317	0.68	0.19	8708	0.80	0.27
sce	19400	0.73	0.20	17295	0.76	0.22	10644	0.83	0.28

Table S10. Most specific existing and predicted functional annotation (per GO subontology) for *MLH1*. Predictions with supporting literature are marked as (+). The correctness of predictions without supporting literature (?) remains unclear.

	Molecular Function	Evidence	Biological Process	Evidence	Cellular Component	Evidence
**existing annotations**	single-stranded DNA binding<br>MutSα complex binding		mismatch repair		nucleus	
**predicted annotations**	ATPase activity	+ (Ban and Yang, 1998; Hall *et al.*, 2002)	base-excision repair	+ (Wu and Vasquez, 2008)	MutLα complex	+ (Jiricny, 2006)
	ATP binding	+ (Ban and Yang, 1998; Hall *et al.*, 2002)	postreplication repair	+ (Shcherbakova *et al.*, 2001)		
	protein homodimerization activity	+ (Shcherbakova *et al.*, 2001)	maintenance of DNA repeat elements	+ (Lin and Wilson, 2009)		
	four-way junction DNA binding	+(Baker *et al.*, 1996)				
	guanine/thymine mispair binding	+ (Yoshioka *et al.*, 2006)				
	dinucleotide repeat insertion binding	?				
	single guanine insertion binding	?				
	purine-specific mismatch base pair DNA N-glycosylase activity	?				
	single thymine insertion binding	?				
	oxidized DNA binding	?				

Table S11. Most specific existing and predicted functional annotation (per GO subontology) for *PMS2*. Predictions with supporting literature are marked as (+). (–) indicates false predictions and for predictions without supporting literature (?) the correctness remains unclear.

	Molecular Function	Evidence	Biological Process	Evidence	Cellular Component	Evidence
**existing annotations**	single-stranded DNA binding<br>single base insertion or deletion binding<br>MutSα complex binding		mismatch repair		nucleus	
**predicted annotations**	ATPase activity<br>ATP binding<br>protein homodimerization activity<br>magnesium ion binding<br>dinucleotide insertion or deletion binding<br>loop DNA binding<br>four-way junction DNA binding<br>guanine/thymine mispair binding<br>single guanine insertion binding<br>purine-specific mismatch base pair DNA N-glycosylase activity<br>single thymine insertion binding<br>oxidized purine DNA binding	+ (Ban and Yang, 1998; Guarne *et al.*, 2001; Hall *et al.*, 2002)<br>+ (Ban and Yang, 1998; Hall *et al.*, 2002)<br>+ (Gibson *et al.*, 2006)<br>+ (Hsieh and Yamane, 2008)<br>+ (Habraken *et al.*, 1997)<br>+ (Habraken *et al.*, 1997)<br>?<br>?<br>?<br>?<br>?<br>?	DNA recombination<br>base-excision repair<br>meiotic mismatch repair	+ (Stone and Petes, 2006; Erdeniz *et al.*, 2007)<br>+ (Wu and Vasquez, 2008)<br>+ (Stone and Petes, 2006; Erdeniz *et al.*, 2007)	MutLα complex	+ (Jiricny, 2006)

Table S12. Most specific existing and predicted functional annotation (per GO subontology) for *EPHB4*. Predictions with supporting literature are marked as (+). (–) indicates false predictions and for predictions without supporting literature (?) the correctness remains unclear.

	Molecular Function	Evidence	Biological Process	Evidence	Cellular Component	Evidence
**existing annotations**	transmembrane receptor protein tyrosine kinase activity		cell proliferation		integral to plasma membrane	
			regulation of angiogenesis		cell surface	
**predicted annotations**	ATP binding	+ (Schlessinger, 2000; Pawson, 2002)	protein amino acid phosphorylation	+ (Schlessinger, 2000; Pawson, 2002)		
	protein binding	+ (Schlessinger, 2000; Pawson, 2002)	behavior	+ (Pasquale, 2005)		
	enzyme regulator activity	+ (Schlessinger, 2000; Pawson, 2002)	cell-cell signaling	+ (Himanen and Nikolov, 2003)		
	protein serine/threonine kinase activity	–	cell migration	+ (Sturz *et al.*, 2004)		
	enzyme binding	+ (Schlessinger, 2000; Pawson, 2002)	transmembrane receptor protein tyrosine kinase signaling pathway	+ (Tanaka *et al.*, 2004)		
	protein C-terminus binding	+ (Schlessinger, 2000)	axon guidance	+ (Brambilla and Klein, 1995; Dickson, 2002; Huot, 2004)		
	SH3/SH2 adaptor activity	+ (Schlessinger and Lemmon, 2003)				
	axon guidance receptor activity	+ (Brambilla and Klein, 1995; Dickson, 2002; Huot, 2004)				
	transmembrane-ephrin receptor activity	+(Ikegaki *et al.*, 1995; Birgbauer *et al.*, 2001)				

References

Altaf-Ul-Amin M, Shinbo Y, Mihara K, Kurokawa K, and Kanaya S (2006). Development and implementation of an algorithm for detection of protein complexes in large interaction networks. *BMC Bioinformatics*, **7**, 207.

Bader GD and Hogue CWV (2003). An automated method for finding molecular complexes in large protein interaction networks. *BMC Bioinformatics*, **4**, 2.

Bader GD, Betel D, and Hogue CWV (2003). BIND: the Biomolecular Interaction Network Database. *Nucleic Acids Res*, **31**(1), 248–250.

Baker SM, Plug AW, Prolla TA, Bronner CE, Harris AC, Yao X, Christie DM, Monell C, Arnheim N, Bradley A, *et al.* (1996). Involvement of mouse Mlh1 in DNA mismatch repair and meiotic crossing over. *Nat Genet*, **13**(3), 336–342.

Ban C and Yang W (1998). Crystal structure and ATPase activity of MutL: implications for DNA repair and mutagenesis. *Cell*, **95**(4), 541–552.

Bieri T, Blasiar D, Ozersky P, Antoshechkin I, Bastiani C, Canaran P, Chan J, Nansheng C, Chen WJ, Davis P, *et al.* (2007). WormBase: new content and better access. *Nucleic Acids Res*, **35**(Database issue), D506–D510.

Birgbauer E, Oster SF, Severin CG, and Sretavan DW (2001). Retinal axon growth cones respond to EphB extracellular domains as inhibitory axon guidance cues. *Development*, **128**(15), 3041–3048.

Boeckmann B, Bairoch A, Apweiler R, Blatter MC, Estreicher A, Gasteiger E, Martin MJ, Michoud K, O'Donovan C, Phan I, *et al.* (2003). The SWISS-PROT protein knowledgebase and its supplement TrEMBL in 2003. *Nucleic Acids Res*, **31**(1), 365–370.

Brambilla R and Klein R (1995). Telling axons where to grow: a role for Eph receptor tyrosine kinases in guidance. *Mol Cell Neurosci*, **6**(6), 487–495.

Bult CJ, Eppig JT, Kadin JA, Richardson JE, Blake JA, and Group MGD (2008). The Mouse Genome Database (MGD): mouse biology and model systems. *Nucleic Acids Res*, **36**(Database issue), D724–D728.

Chatr-aryamontri A, Ceol A, Montecchi-Palazzi L, Nardelli G, Schneider MV, Castagnoli L, and Cesareni G (2007). MINT: the Molecular INTeraction database. *Nucleic Acids Research*, **35**(Database-Issue), 572–574.

Chua HN, Sung WK, and Wong L (2006). Exploiting indirect neighbours and topological weight to predict protein function from protein-protein interactions. *Bioinformatics*, **22**(13), 1623–1630.

Chua HN, Sung WK, and Wong L (2007). Using indirect protein interactions for the prediction of gene ontology functions. *BMC Bioinformatics*, **8 Suppl 4**, S8.

Couto FM, Silva MJ, and Pedro Coutinho PM (2007). Measuring semantic similarity between gene ontology terms. *Data Knowl. Eng*, **61**(1), 137–152.

Dickson BJ (2002). Molecular mechanisms of axon guidance. *Science*, **298**(5600), 1959–1964.

Erdeniz N, Nguyen M, Deschenes SM, and Liskay RM (2007). Mutations affecting a putative mutlα endonuclease motif impact multiple mismatch repair functions. *DNA Repair (Amst)*, **6**(10), 1463–1470.

FlyBase Consortium (2003). The FlyBase database of the drosophila genome projects and community literature. *Nucleic Acids Res*, **31**(1), 172–175.

Freedman D, Pisani R, and Purves R (1998). *Statistics*. New York: W.W. Norton and Company, 3 edition.

Gavin AC, Bsche M, Krause R, Grandi P, Marzioch M, Bauer A, Schultz J, Rick JM, Michon AM, Cruciat CM, Remor M, Hfert C, Schelder M, Brajenovic M, Ruffner H, Merino A, Klein K, Hudak M, Dickson D, Rudi T, Gnau V, Bauch A, Bastuck S, Huhse B, Leutwein C, Heurtier MA, Copley RR, Edelmann A, Querfurth E, Rybin V, Drewes G, Raida M, Bouwmeester T, Bork P, Seraphin B, Kuster B, Neubauer G, and Superti-Furga G (2002). Functional organization of the yeast proteome by systematic analysis of protein complexes. *Nature*, **415**(6868), 141–147.

Gibson SL, Narayanan L, Hegan DC, Buermeyer AB, Liskay RM, and Glazer PM (2006). Overexpression of the DNA mismatch repair factor, PMS2, confers hypermutability and dna damage tolerance. *Cancer Lett*, **244**(2), 195–202.

Giot L, Bader JS, Brouwer C, Chaudhuri A, Kuang B, Li Y, Hao YL, Ooi CE, Godwin B, Vitols E, *et al.* (2003). A protein interaction map of drosophila melanogaster. *Science*, **302**(5651), 1727–1736.

Guarne A, Junop MS, and Yang W (2001). Structure and function of the N-terminal 40 kDa fragment of human PMS2: a monomeric GHL ATPase. *EMBO J*, **20**(19), 5521–5531.

Habraken Y, Sung P, Prakash L, and Prakash S (1997). Enhancement of MSH2-MSH3-mediated mismatch recognition by the yeast MLH1-PMS1 complex. *Curr Biol*, **7**(10), 790–793.

Hall MC, Shcherbakova PV, and Kunkel TA (2002). Differential ATP binding and intrinsic ATP hydrolysis by amino-terminal domains of the yeast Mlh1 and Pms1 proteins. *J Biol Chem*, **277**(5), 3673–3679.

Hermjakob H, Montecchi-Palazzi L, Lewington C, Mudali S, Kerrien S, Orchard S, Vingron M, Roechert B, Roepstorff P, Valencia A, *et al.* (2004). IntAct: an open source molecular interaction database. *Nucleic Acids Res*, **32**(Database issue), D452–D455.

Himanen JP and Nikolov DB (2003). Eph receptors and ephrins. *Int J Biochem Cell Biol*, **35**(2), 130–134.

Hong EL, Balakrishnan R, Dong Q, Christie KR, Park J, Binkley G, Costanzo MC, Dwight SS, Engel SR, Fisk DG, *et al.* (2008). Gene ontology annotations at SGD: new data sources and annotation methods. *Nucleic Acids Res*, **36**(Database issue), D577–D581.

Hsieh P and Yamane K (2008). DNA mismatch repair: molecular mechanism, cancer, and ageing. *Mech Ageing Dev*, **129**(7-8), 391–407.

Huot J (2004). Ephrin signaling in axon guidance. *Prog Neuropsychopharmacol Biol Psychiatry*, **28**(5), 813–818.

Ikegaki N, Tang XX, Liu XG, Biegel JA, Allen C, Yoshioka A, Sulman EP, Brodeur GM, and Pleasure DE (1995). Molecular characterization and chromosomal localization of DRT (EPHT3): a developmentally regulated human protein-tyrosine kinase gene of the EPH family. *Hum Mol Genet*, **4**(11), 2033–2045.

Jiricny J (2006). MutLalpha: at the cutting edge of mismatch repair. *Cell*, **126**(2), 239–241.

Lehner B and Fraser AG (2004). A first-draft human protein-interaction map. *Genome Biol*, **5**(9), R63.

Lin D (1998). An information-theoretic definition of similarity. In *Proceedings of the 15th ICML*, pages 296–304, Madison WI.

Lin Y and Wilson JH (2009). Diverse effects of individual mismatch repair components on transcription-induced CAG repeat instability in human cells. *DNA Repair (Amst)*, **8**(8), 878–885.

Mulder NJ and Apweiler R (2008). The InterPro database and tools for protein domain analysis. *Curr Protoc Bioinformatics*, **Chapter 2**, Unit 2.7.

Pagel P, Kovac S, Oesterheld M, Brauner B, Dunger-Kaltenbach I, Frishman G, Montrone C, Mark P, Stümpflen V, Mewes HW, *et al.* (2005). The MIPS mammalian protein-protein interaction database. *Bioinformatics*, **21**(6), 832–834.

Pasquale EB (2005). Eph receptor signalling casts a wide net on cell behaviour. *Nat Rev Mol Cell Biol*, **6**(6), 462–475.

Pawson T (2002). Regulation and targets of receptor tyrosine kinases. *Eur J Cancer*, **38 Suppl 5**, S3–10.

Peri S, Navarro JD, Amanchy R, Kristiansen TZ, Jonnalagadda CK, Surendranath V, Niranjan V, Muthusamy B, Gandhi TKB, Gronborg M, *et al.* (2003). Development of human protein reference database as an initial platform for approaching systems biology in humans. *Genome Res*, **13**(10), 2363–2371.

Rual JF, Venkatesan K, Hao T, Hirozane-Kishikawa T, Dricot A, Li N, Berriz GF, Gibbons FD, Dreze M, Ayivi-Guedehoussou N, Klitgord N, Simon C, Boxem M, Milstein S, Rosenberg J, Goldberg DS, Zhang LV, Wong SL, Franklin G, Li S, Albala JS, Lim J, Fraughton C, Llamosas E, Cevik S, Bex C, Lamesch P, Sikorski RS, Vandenhaute J, Zoghbi HY, Smolyar A, Bosak S, Sequerra R, Doucette-Stamm L, Cusick ME, Hill DE, Roth FP, and Vidal M (2005). Towards a proteome-scale map of the human protein-protein interaction network. *Nature*, **437**(7062), 1173–1178.

Salwinski L, Miller CS, Smith AJ, Pettit FK, Bowie JU, and Eisenberg D (2004). The Database of Interacting Proteins: 2004 update. *Nucleic Acids Res*, **32**(Database issue), D449–D451.

Schlessinger J (2000). Cell signaling by receptor tyrosine kinases. *Cell*, **103**(2), 211–225.

Schlessinger J and Lemmon MA (2003). SH2 and PTB domains in tyrosine kinase signaling. *Sci STKE*, **2003**(191), RE12.

Sharan R, Suthram S, Kelley RM, Kuhn T, McCuine S, Uetz P, Sittler T, Karp RM, and Ideker T (2005). Conserved patterns of protein interaction in multiple species. *Proc Natl Acad Sci U S A*, **102**(6), 1974–1979.

Shcherbakova PV, Hall MC, Lewis MS, Bennett SE, Martin KJ, Bushel PR, Afshari CA, and Kunkel TA (2001). Inactivation of DNA mismatch repair by increased expression of yeast MLH1. *Mol Cell Biol*, **21**(3), 940–951.

Spirin V and Mirny LA (2003). Protein complexes and functional modules in molecular networks. *Proc Natl Acad Sci U S A*, **100**(21), 12123–12128.

Stark C, Breitkreutz BK, Reguly T, Boucher L, Breitkreutz A, and Tyers M (2006). BioGRID: a general repository for interaction datasets. *Nucleic Acids Research*, **34**(Database-Issue), 535–539.

Stelzl U, Worm U, Lalowski M, Haenig C, Brembeck FH, Goehler H, Stroedicke M, Zenkner M, Schoenherr A, Koeppen S, Timm J, Mintzlaff S, Abraham C, Bock N, Kietzmann S, Goedde A, Toksz E, Droege A, Krobitsch S, Korn B, Birchmeier W, Lehrach H, and Wanker EE (2005). A human protein-protein interaction network: a resource for annotating the proteome. *Cell*, **122**(6), 957–968.

Stone JE and Petes TD (2006). Analysis of the proteins involved in the in vivo repair of base-base mismatches and four-base loops formed during meiotic recombination in the yeast saccharomyces cerevisiae. *Genetics*, **173**(3), 1223–1239.

Sturz A, Bader B, Thierauch KH, and Glienke J (2004). EphB4 signaling is capable of mediating ephrinB2-induced inhibition of cell migration. *Biochem Biophys Res Commun*, **313**(1), 80–88.

Tanaka M, Ohashi R, Nakamura R, Shinmura K, Kamo T, Sakai R, and Sugimura H (2004). Tiam1 mediates neurite outgrowth induced by ephrin-B1 and EphA2. *EMBO J*, **23**(5), 1075–1088.

Twigger SN, Shimoyama M, Bromberg S, Kwitek AE, Jacob HJ, and Team RGD (2007). The Rat Genome Database, update 2007–easing the path from disease to data and back again. *Nucleic Acids Res*, **35**(Database issue), D658–D662.

Uetz P, Giot L, Cagney G, Mansfield TA, Judson RS, Knight JR, Lockshon D, Narayan V, Srinivasan M, Pochart P, Qureshi-Emili A, Li Y, Godwin B, Conover D, Kalbfleisch T, Vijayadamodar G, Yang M, Johnston M, Fields S, and Rothberg JM (2000). A comprehensive analysis of protein-protein interactions in saccharomyces cerevisiae. *Nature*, **403**(6770), 623–627.

Walhout AJ and Vidal M (2001). High-throughput yeast two-hybrid assays for large-scale protein interaction mapping. *Methods*, **24**(3), 297–306.

Wheeler DL, Barrett T, Benson DA, Bryant SH, Canese K, Chetvernin V, Church DM, Dicuccio M, Edgar R, Federhen S, *et al.* (2008). Database resources of the National Center for Biotechnology Information. *Nucleic Acids Res*, **36**(Database issue), D13–D21.

Wu Q and Vasquez KM (2008). Human MLH1 protein participates in genomic damage checkpoint signaling in response to DNA interstrand crosslinks, while MSH2 functions in dna repair. *PLoS Genet*, **4**(9), e1000189.

Yoshioka K, Yoshioka Y, and Hsieh P (2006). ATR kinase activation mediated by MutSalpha and MutLalpha in response to cytotoxic O6-methylguanine adducts. *Mol Cell*, **22**(4), 501–510.
